# A high-throughput screening platform for Polycystic Kidney Disease (PKD) drug repurposing utilizing murine and human ADPKD cells

**DOI:** 10.1038/s41598-020-61082-3

**Published:** 2020-03-06

**Authors:** Rosita R. Asawa, Carina Danchik, Alexey Zakharov, Yuchi Chen, Ty Voss, Ajit Jadhav, Darren P. Wallace, Josephine F. Trott, Robert H. Weiss, Anton Simeonov, Natalia J. Martinez

**Affiliations:** 1 National Center for Advancing Translational Sciences, National Institutes of Health, Rockville, MD USA; 2 Department of Internal Medicine, University of Kansas Medical Center, Kansas City, KS USA; 3 Division of Nephrology, Department of Internal Medicine, University of California, Davis, CA USA

**Keywords:** High-throughput screening, Polycystic kidney disease, Phenotypic screening

## Abstract

Autosomal dominant polycystic kidney disease (ADPKD) is one of the most common inherited monogenic disorders, characterized by a progressive decline in kidney function due in part to the formation of fluid-filled cysts. While there is one FDA-approved therapy, it is associated with potential adverse effects, and all other clinical interventions are largely supportive. Insights into the cellular pathways underlying ADPKD have revealed striking similarities to cancer. Moreover, several drugs originally developed for cancer have shown to ameliorate cyst formation and disease progression in animal models of ADPKD. These observations prompted us to develop a high-throughput screening platform of cancer drugs in a quest to repurpose them for ADPKD. We screened ~8,000 compounds, including compounds with oncological annotations, as well as FDA-approved drugs, and identified 155 that reduced the viability of *Pkd1*-null mouse kidney cells with minimal effects on wild-type cells. We found that 109 of these compounds also reduced *in vitro* cyst growth of *Pkd1-null* cells cultured in a 3D matrix. Moreover, the result of the cyst assay identified therapeutically relevant compounds, including agents that interfere with tubulin dynamics and reduced cyst growth without affecting cell viability. Because it is known that several ADPKD therapies with promising outcomes in animal models failed to be translated to human disease, our platform also incorporated the evaluation of compounds in a panel of primary ADPKD and normal human kidney (NHK) epithelial cells. Although we observed differences in compound response amongst ADPKD and NHK cell preparation, we identified 18 compounds that preferentially affected the viability of most ADPKD cells with minimal effects on NHK cells. Our study identifies attractive candidates for future efficacy studies in advanced pre-clinical models of ADPKD.

## Introduction

Autosomal dominant polycystic kidney disease (ADPKD) has a prevalence of 1 in 500–1,000 individuals and is the most common inherited kidney disease, accounting for 6–9% of patients on renal replacement therapy. ADPKD is caused mainly by mutations in *PKD1* and to a lesser extent in *PKD2, GANAB* and *DNAJB11* genes. The disease is characterized by a progressive decline in kidney function due to the formation of fluid-filled cysts as well as activation of inflammatory and proliferative pathways, typically leading to end-stage renal disease by the fifth or sixth decade of life^1^. Although cyst formation in the kidneys is the hallmark of ADPKD, other epithelial organs including the liver and pancreas are also commonly affected^2,3^. With the 2018 FDA approval of Tolvaptan, a vasopressin V_2_-receptor antagonist, there is now one therapy available to slow disease progression; however, the drug was only approved for patients at risk of rapid disease progression due to its potential side effects. Nevertheless, most interventions focus on alleviating disease-related symptoms.

Research on the signaling pathways and pathological disorders underlying ADPKD has revealed that many of the same metabolic pathways associated with epithelial proliferation, apoptosis, and extracellular matrix remodeling are shared between cancer and cystic disease^4^. In recent years, the utility of investigating these parallels has been exploited such that available cancer drugs can be repurposed to treat ADPKD. For example, the p21 activated kinase (PAK)/WNT/β-catenin pathway, the AMP-activated protein kinase (AMPK) pathways, glucose metabolism and the microtubule cytoskeleton, are all potential targets for ADPKD. Correspondingly, PAK-4 inhibition with KPT-9274, AMPK activation with Metformin, glycolysis inhibition with the glucose analog 2-deoxy-D-glucose, and microtubule depolymerization inhibition with Taxol, have all been shown to attenuate cyst formation and ADPKD progression in murine models^5–9^.

To facilitate the repurposing of effective cancer drugs for use in ADPKD patients, we sought to establish a high-throughput screening platform. We have previously shown that PAK4 inhibition with KPT-9274 preferentially reduces the viability of *Pkd1*-null over wild-type (wt) cells^5^. Based on these findings, we employed a phenotypic screening paradigm using mouse embryonic as well as postnatal *Pkd1*-null kidney cells to evaluate a collection of 8,814 unique compounds spanning approved drugs and investigational agents, the majority of which have antineoplastic indications^10,11^. We identified a set of compounds that reduced the viability of *Pkd1*-null cells but had minimal effects on wt cells. To further investigate the therapeutic potential of this set of compounds, we developed a high-throughput three-dimensional (3D) assay to assess the effect of compounds on cyst growth *in vitro*. Finally, we profiled the set of compounds for their effect on the viability of primary human ADPKD and normal epithelial kidney (NHK) cells. Overall, our platform enables the systematic identification of chemical agents with preferential activity in ADPKD over normal cells. These agents constitute attractive candidates for future efficacy studies in pre-clinical models of ADPKD.

## Results

### A phenotypic quantitative high-throughput screen (qHTS) identifies compounds preferentially affecting the viability of *Pkd1*-null vs. wt epithelial kidney cells

**Figure 1:**
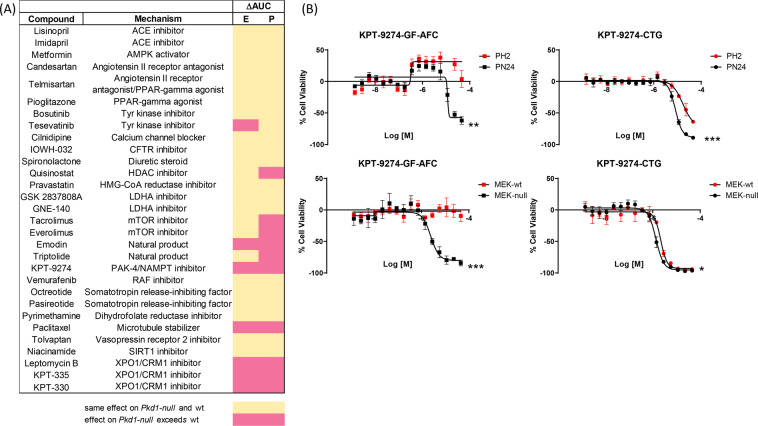
Cell viability outcome of reference set compounds. (**A**) Differential response between *Pkd1*-null and wt cells is shown as difference in area under the curve (ΔAUC) for mouse embryonic (E) and postnatal (P) kidney cell pairs. Pink: differential response between *Pkd1*-null vs. wt; yellow: no difference between *Pkd1*-null vs. wt/no response. (**B**) PAK-4 inhibitor KPT-9274 reduces the viability of *Pkd1*-null cells. Dose response curves of KPT-9274 treated postnatal (top) and MEK (bottom) *Pkd1*-null (black) and wt (red) cell pairs. Responses obtained with the GF-AFC and CTG reagents are shown on the left and right, respectively. Data is represented as Mean ± SD, n = 3. Unpaired, parametric (mean AUC) T-test (Welch’s correction) *p-val < 0.05; **p-val < 0.001; ***p-val < 0.0001.

We have previously shown that PAK4 inhibition via treatment with KPT-9274, a PAK4/NAMPT dual inhibitor, causes reduction of cell proliferation and induction of apoptosis in *Pkd1*-null cells as well as reduction of cystogenesis both *ex vivo* and *in vivo*^5^. Specifically, we showed that KPT-9274 was more effective at reducing the proliferation of *Pkd1*-null postnatal cells of proximal tubule origin (PN24) and *Pkd1*-null mouse embryonic kidney cells of collecting duct origin (MEK-null) than that of control *Pkd1*-heterozygous (PH2) and *Pkd1*-wild type MEK (MEK-wt) cells, respectively^5^. Based on these findings, we established a quantitative high-throughput screen (qHTS) to identify small molecules that preferentially reduce the viability/proliferation of *Pkd1*-null cells (PN24 and MEK-null) compared to their wt counterparts (PH2 and MEK-wt). The assay was performed in a 1,536-well format and measured viable cells after 48 hours of compound treatment. Specifically, the viability readout was obtained first by addition of the pro-fluorescent Gly-Phe-7-Amino-4-Trifluoromethylcoumarin (GF-AFC) peptide, which is cleaved by cell-membrane bound proteases present in intact viable cells, thus producing fluorescence^12^, followed by addition of the luminescence-based CellTiter-Glo (CTG) reagent, which measures cellular ATP levels. It is important to note that all cell lines actively proliferated during the time course of the assay (Supplementary Fig. 1). qHTS paradigms allow the generation of concentration-response curves (CRCs) directly from the primary screen and both potency and efficacy, as well as Area Under the dose-response Curve (AUC) values, can be used to identify compounds with robust bioactivity profiles^13,14^. As proof of principle, we compiled a collection of reference compounds, or analogs thereof, previously tested in pre-clinical and clinical trials of PKD^5,9,15–21^ (Fig. 1A). This reference set contained 30 compounds and span 22 different targets/mode of action (MOA). We found that 11 compounds (9 targets/MOA) displayed differences in AUC values (referred to as ΔAUC) in at least one pair of *Pkd1*-null vs. wt cells. For instance, the mTOR inhibitors Tracolimus and Everolimus, the natural product Triptolide and the HDAC inhibitor Quisinostat all preferentially reduced the viability of PN24 vs. PH2 cells but showed no difference between MEK-null and MEK-wt cells. Conversely, the kinase inhibitor Tesevatinib, preferentially reduced the viability of MEK-null vs. MEK-wt cells but showed no difference between PN24 vs PH2 cells. On the other hand, XPO1/CRM1 inhibitors Leptomycin B, KPT-335 and KPT-330, the natural product Emodin, and the microtubule stabilizer Paclitaxel, decreased the viability of *Pkd1*-null vs. wt cells in both postnatal and embryonic kidney cell pairs (Fig. 1A; Supplementary Fig. 2). Interestingly, in the GF-AFC readout KPT-9274 reduced the viability of both PN24 and MEK-null cells with minimal effects on wt cells (Fig. 1B). However, ATP levels as measured by CTG were similarly reduced in all cell lines after KPT-9274 treatment. The remaining compounds showed either no effect on cell viability or equally reduced the viability of wt and *Pkd1*-null cells. It is not surprising that among the inactive compounds we found diuretics, ACE inhibitors, Angiotensin Receptor Blockers and Calcium channel blockers, which are indicated for the treatment of ADPKD-associated hypertension and are not expected to reduce cell proliferation. Other compounds that failed to elicit a differential effect in cell proliferation includes agents that reduce fluid secretion by indirectly blocking CFTR function (CFTR and PPARy inhibitors) or cAMP activation (Vasopressin Receptor inhibitors and Somatostatin analogs), which were previously shown to reduce cell proliferation in *in vivo* models of ADPKD^22–25^. However, in contrast to *in vivo* models, the effect of Tolvaptan and CFTR inhibitors on *in vitro* cell proliferation requires stimulation of elevated intracellular cAMP levels^22,26^. These observations are in agreement with our findings using MEK and PN/PH cells, since proliferation assays were carried out in the absence of cAMP stimulants. The PPARy agonist Pioglitazone was shown to reduce cyst size *in vivo*^23^, and to our knowledge investigation of Pioglitazone effects in *in vitro* cell cultures has not been reported. Similarly, we found that AMPK activation via Metformin had no effect on cell proliferation. Although Metformin was suggested to have beneficial effects on disease progression^27^, the effect of Metformin on cell proliferation and cyst growth has been brought into question by another recent study^28^. Additionally, differences in concentration or time frame of compound treatment could account for the lack of activity observed. For example, the anti-parasitic Pyrimethamine has been shown to decrease cell proliferation of human ADPKD cells at concentrations higher than those achieved in our assay^18^. Similarly, Niacinamide was previously used at mM concentrations to inhibit Sirtuin 1 activity^29^, while we used it at µM concentrations. Finally, the specific compound we used to modulate a given pathway did not always exactly match the compound used in reported studies. For example, inhibition of glycolysis with 2-deoxy-D-glucose (2DG) has been shown to reduce cell proliferation in models of ADPKD^8,21^, but blockage of lactate production with LDHA inhibitors did not lead to reduction of cell proliferation in our assay. However, to our knowledge, LDHA inhibitors have not been tested in ADPKD models before. Altogether, our results indicate that the cellular proliferation responses observed using the compounds in the reference set agree with previous findings and several of the compounds in the set elicit growth/viability differences between *Pkd1*-null and wt cells and can be identified using the screening paradigm presented here. Hence, this strategy constitutes a viable avenue to identify chemical agents for potential repurposing for ADPKD.

**Figure 2:**
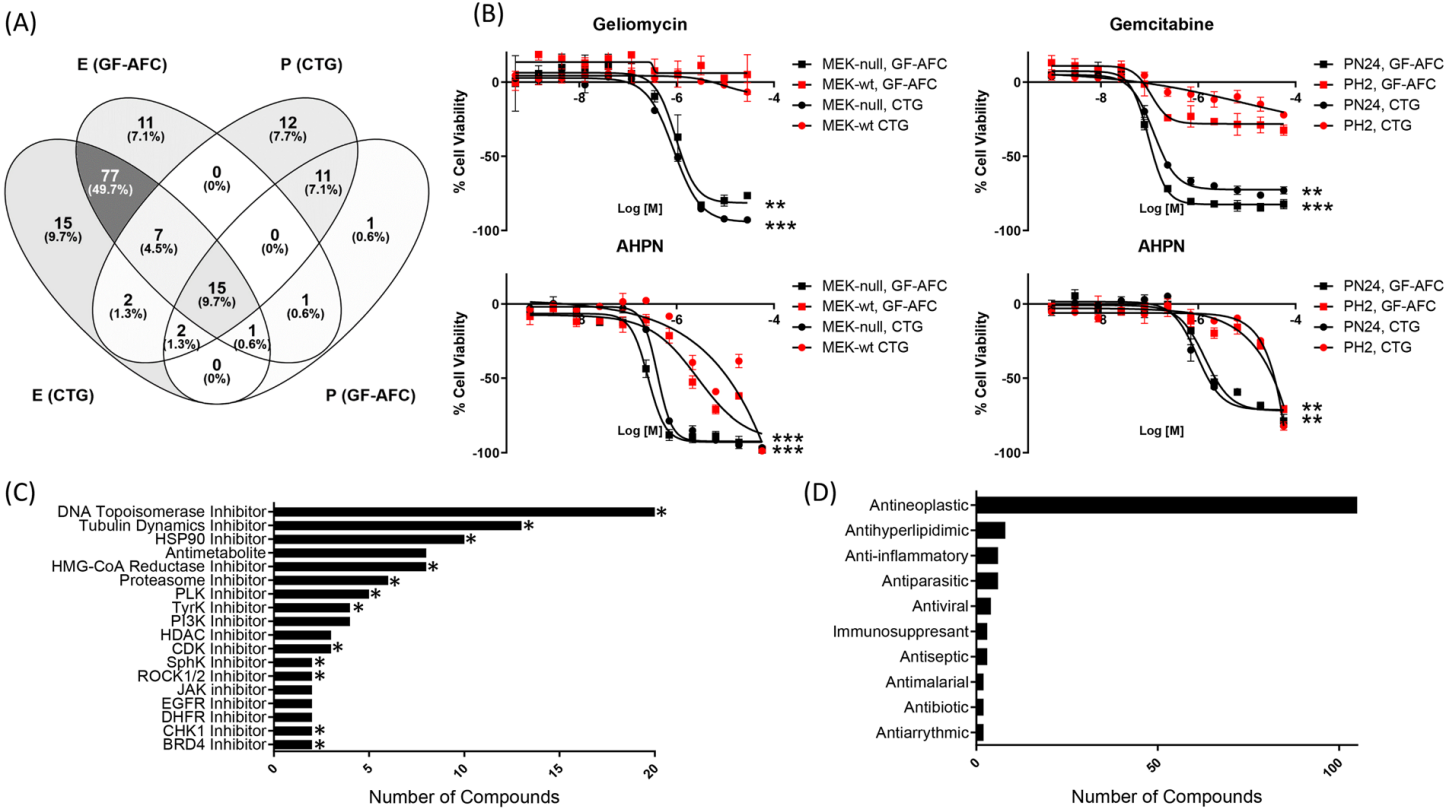
qHTS identifies multiple compounds with differential viability effects in *Pkd1*-null vs. wt cells. (**A**) Venn diagram shows the number of unique compounds with differential viability effects identified in each cell pair and each readout. E-embryonic and P-postnatal kidney cells. (**B**) Examples of compounds showing differential effects in only embryonic (Geliomycin, top left), only postnatal (Gemcitabine, top right) or in both cell models (AHPN, bottom). Data is represented as Mean ± SD, n = 3. Unpaired, parametric (mean AUC) T-test (Welch’s correction) *p-val < 0.05; **p-val < 0.001; ***p-val < 0.0001. (**C**) Target-based analysis of compounds with differential activity in *Pkd1*-null vs. wt cells. Asterisks indicate statistically significant enrichment of target classes. Only target classes containing two or more compounds are shown. Note that for Antimetabolites enrichment analysis is not possible due to lack of library annotation for this category. (**D**) Primary Indication of compounds with differential activity in *Pkd1*-null vs. wt cells.

To identify new therapeutic options for PKD, we screened 8,814 unique compounds using the screening paradigm described above. The small molecule collections we chose included the NIH Chemical Genomics Center (NCGC) Pharmaceutical Collection (NPC), Mechanism of Interrogation Plate (MIPE), the NCATS Pharmacologically Active Chemical Toolbox (NPACT), and the Kinase Inhibitor collections, comprised of approved and investigational drugs as well as highly-annotated tool compounds with varying degrees of validation as therapeutics^10,11,30–33^. For all compounds tested, we derived CRCs in all four cell lines and we used ΔAUC values to identify compounds with robust bioactivity profiles against *Pkd1*-null cells but minimal or less pronounced effects in wt cells (activity cutoffs are described in Materials and Methods and assay performance is described in Supplementary Table 1). A total of 210 unique compounds showed differential activity against at least one pair of cell lines in at least one readout and were selected for confirmation studies (Supplementary Table 2). Validation assays, in which fresh compound solutions were plated at 11 doses, confirmed the activity of 155 unique compounds (Supplementary Table 3). Among confirmed compounds, we found that ~18% showed a differential response in both embryonic and postnatal cell pairs in either one or both readouts. While ~15% of the compounds showed differential responses in postnatal cells only, the vast majority (~66%) of compounds showed a differential response in embryonic but not in postnatal kidney cells (Fig. 2A,B). These observations could be explained by the differential underlying biology and different origin between embryonic and postnatal kidney cells: while PH2/PN24 epithelial cells are derived from adult proximal tubules, MEK epithelial cells are derived from the collecting ducts of embryonic kidneys, which comprise both intercalated and principal cells^34^. In addition, these two models differ in their *Pkd1* gene dosage; in the embryonic model, we are comparing *Pkd1-*null (MEK-null) with *Pkd1*-wt (MEK-wt) cells, while in the postnatal model we are comparing *Pkd1*-null (PN24) with *Pkd1*-heterozygous cells (PH2), with only one functional copy of *Pkd1*. Differential activity of ~73% of compounds was detected by both GF-AFC and CTG readouts, indicating good agreement between readouts. Among active compounds, our screen identified HMG-CoA Reductase, HSP90, Tubulin Depolymerization, Tyrosine Kinase, XPO1/CRM, CDK, BRD4 and HDAC inhibitors as having differential responses between *Pkd1*-null and wt cells (Supplementary Table 4). These inhibitors regulate cellular mechanisms and pathways previously shown to play a role in cystic disease progression, and many of them have been proposed as therapeutic options for ADPKD^4,9,16,17,35–37^. Additionally, we found proteasome inhibitors (Supplementary Table 4) which have been shown to reduce cystic disease in models of human autosomal dominant polycystic liver disease (ADPLD)^38^. An unbiased target-based analysis of the pharmacological responses of the 155 hits indicated a statistically significant enrichment of several inhibitor classes, including some of the aforementioned classes (Fig. 2C). Interestingly, we uncovered enriched classes such as DNA Topoisomerase and Sphingosine Kinase (SphK) inhibitors which, to the best of our knowledge, have not been previously identified as possible therapeutic options for ADPKD. Most hits have antineoplastic indications, which is not surprising given the enrichment of antineoplastic compounds in the libraries (Fig. 2D, Supplementary Table 4).

### A 3D high-throughput assay confirms compound effect on *Pkd1*-null cyst growth

**Figure 3:**
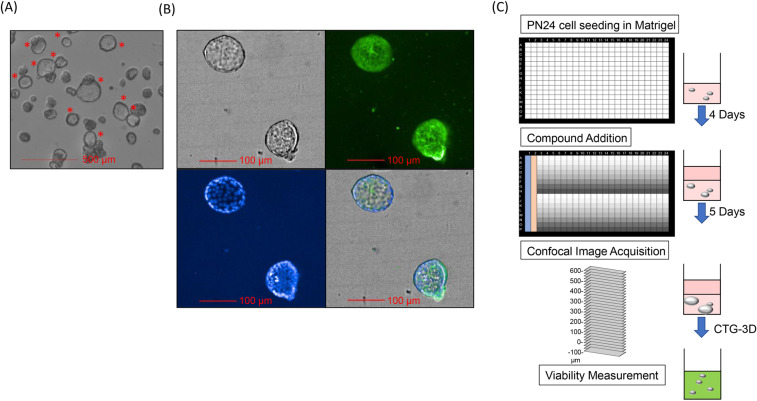
A high-throughput 3D cyst growth assay. (**A**) Bright-field image (representative Z-plane) at 5X magnification of PN24 cysts grown in 40% Matrigel for 9 days. Red asterisks show examples of swelled cysts with a visible lumen. (**B**) Cysts were stained with WGA-Alexa-488 (green; top right) and Hoeschst (blue; bottom left) and imaged at 20X magnification (representative Z-plane is shown). Bright-field (top left) and merged images (bottom right) are also shown. (**C**) Schematic representation of the 384-well 3D cyst assay. PN24 cells were embedded in 40% Matrigel and allowed to form cysts for 4 days. Compound solutions were added following the plate map shown: Columns 1 and 2 contained positive and neutral controls, respectively. Columns 3–24, rows A and I, contained test compounds at 8 column-wise dilution points. Plates were incubated for an additional 5 days before bright-field confocal imaging as in (**A**), followed by addition of CTG-3D to obtain an endpoint measurement of cell viability.

**Figure 4:**
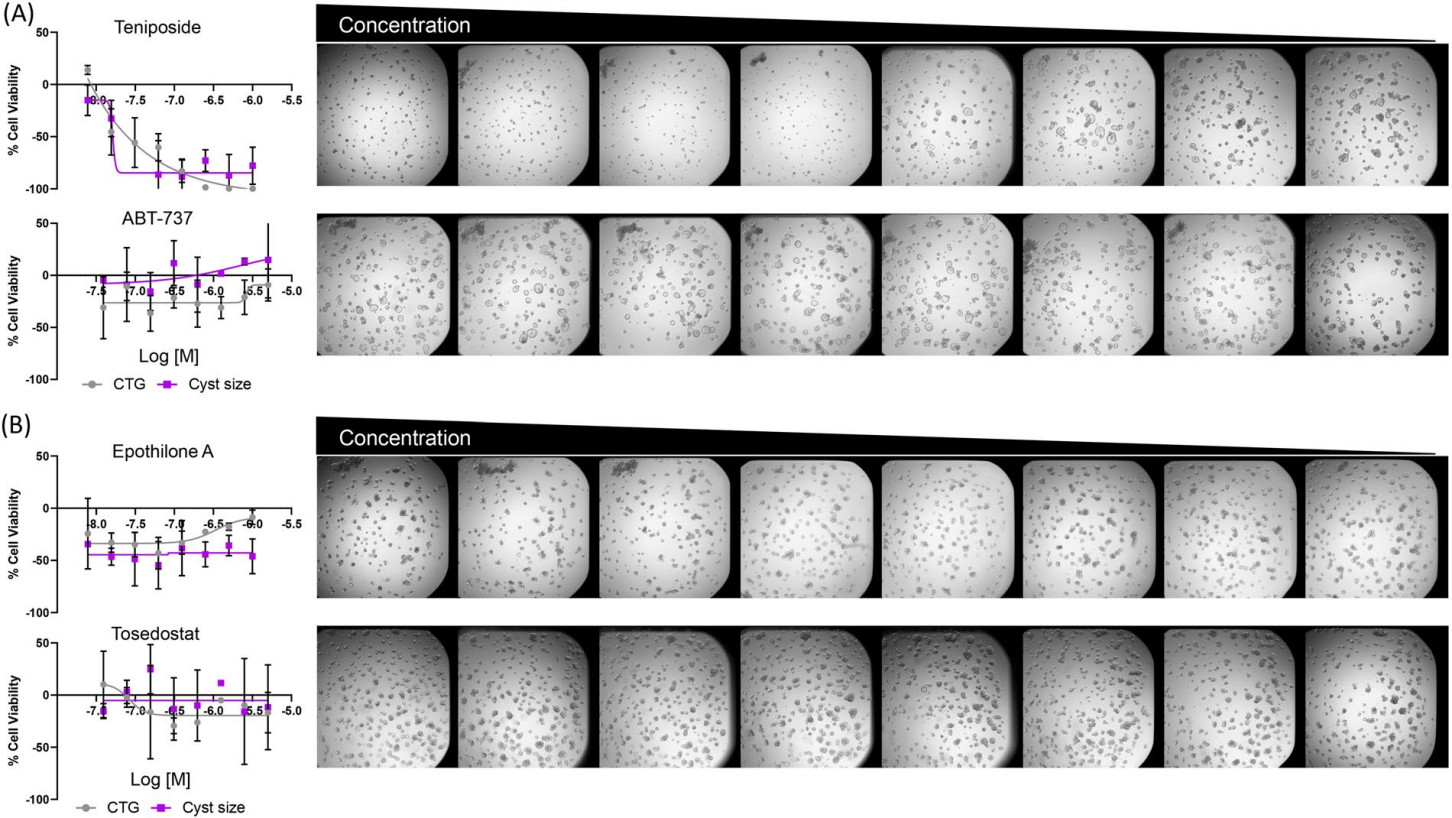
The 3D assay identifies multiple compounds that reduce cyst growth and swelling. (**A**) Examples of compounds that either reduce (Teniposide) or have no effect (ABT-737) on cyst growth. Graphs on the left indicate dose response curves of each compound in the CTG (grey circles) and imaging-based cyst size (purple squares) readouts. Data is represented as Mean ± SD, n = 3. Representative images of wells treated with the indicated compound are on the right. Each compound was tested at a total of 8 concentration points (1:2 serial dilutions). Teniposide was tested at a concentration range of 10–0.078 µM and ABT-737 at 50–0.39 µM. (**B**) Compounds that reduce cyst swelling and have minimal effect on viability. Graphs on the left indicate dose response curves of each compound in the CTG (grey circles) and imaging-based cyst size (purple squares) readouts. Representative images of wells treated with the indicated compound are on the right. Each compound was tested at a total of 8 concentration points (1:2 serial dilutions). Epothilone A was tested at a concentration range of 10–0.078 µM and Tosedostat at 50–0.39 µM.

To identify effective modulators of cyst growth we sought to implement a more physiologically relevant assay. A 3D assay to adequately recapitulate disease pathophysiology is particularly relevant to ADPKD since cysts cannot form when cells are grown in two-dimensional (2D) monolayers. It has been shown that *Pkd1*-null postnatal renal cells form *in vitro* cysts when grown embedded in a 3D-collagen matrix, even in the absence of exogenously added cAMP^39,40^. Accordingly, we found that some PN24 cells swell into cysts with a visible lumen after 4–5 days of culture in 40% Matrigel, while others grew as enlarging cell aggregates with no visible lumen (Fig. 3A,B; Supplementary Fig. 3A,B; Supplementary Movie). In contrast, we did not detect cyst swelling in MEK-null cells as they grew only as enlarging aggregates (Supplementary Fig. 3A). Based on these findings, we used PN24-derived cysts to test the effect of compounds on cyst growth. To profile a large set of compounds in dose response, we seeded PN24 cells onto 384-well plates in 40% Matrigel and first allowed them to form cysts for 4 days before treating them with test compounds for 5 additional days. We then performed confocal imaging and analyzed the images to quantify cyst number and area (Fig. 3C; Supplementary Fig. 3C and Materials and Methods). Of note, our size-based analysis was unable to distinguish between a cell aggregate or swelled cyst with a visible lumen that are of the same size, making visual inspection of images necessary to determine the presence of swelled cysts. Following imaging, we obtained an endpoint measurement of cell viability using the lytic reagent CellTiter-Glo-3D. Using this protocol, we determined optimal cell seeding numbers and maximal tolerated DMSO concentration (Supplementary Fig. 4). We first tested the effect of the 11 compounds from the reference set that showed a differential viability effect between *Pkd1-*null and wt cells in the monolayer viability assay (from Fig. 1A). Treatment with a range of doses of Triptolide, Quisinostat, and the XPO1/CRM inhibitors KPT-335, KPT-330 and Leptomycin B, reduced the cyst size and cell viability compared to DMSO (Supplementary Fig. 5). Paclitaxel and the mTOR inhibitors Tacrolimus and Everolimus had an effect only at high doses, while Emodin and KPT-9274 treatment had no significant effect. Interestingly, Paclitaxel treatment led to reduced cyst swelling even at concentrations where no effect on cyst size and cell viability was detected (Supplementary Fig. 5). We then tested the effect of those 19 compounds in the reference set that did not elicit a differential viability effect between *Pkd1*-null and wt cells. Three of these compounds, Bosutinib, Vermurafenib and the CFTR inhibitor IOWH-032, which equally reduced the viability of both wt and *Pkd1*-null cell types (as ΔAUC did not pass the cutoff), reduced cyst formation. One other compound, the Somatostatin analog Ocreotide, which was inactive in the 2D viability assay, also reduced cyst growth (Supplementary Fig. 6). Of note, Ocreotide reduced cyst growth at concentrations higher than those achieved in the 2D viability assay. The remaining compounds, which were inactive in the 2D viability assay, were also inactive in the 3D assay. Second, we tested the effect of the 155 hits identified in the monolayer screening on cyst growth. While the CTG readout had an excellent average Z’ factor of 0.81, the imaging-based cyst-size readout had a weak average Z’ factor of 0.039, due to the variable proportion of expanding cysts in each well within the DMSO control group (assay statistics provided in Supplementary Table 1). Hence, due to its better assay performance, the CTG-3D readout was used to define activity cutoffs (Materials and Methods). We found that 109 compounds reduced cyst growth while the remaining 46 were either inactive or inconclusive (Supplementary Table 5). Figure 4A displays examples of active (Teniposide) and inactive (ABT-737) compounds (Fig. 4A). Similar to Paclitaxel, other compounds that affect tubulin dynamics such as the antiparasitics Oxibendazole, Flubendazole, Albendazole and Epothilone D, also prevented cyst swelling even at concentration where no effect on viability was observed (Supplementary Fig. 7). We visually inspected the images of compounds that had minimal effects on cell viability and overall cyst size (inactive/inconclusive) to identify additional compounds that reduced cyst swelling. We found that another tubulin depolymerization inhibitor Epothilone A, the ROCK 1/2 inhibitor GSK-269962A, the antimetabolite 5-Azacytidine, and the aminopeptidase inhibitor Tosedostat, visibly reduced cyst swelling at all or most concentrations tested (Fig. 4B and Supplementary Fig. 8).

A high-content cystogenic assay that uses mIMCD3 cells cultured in 3D hydrogels was recently reported^41^. This assay was used to screen a 273-kinase inhibitor library for molecules that block forskolin-induced cyst swelling. Although most of the hits identified in this study were also tested in our initial monolayer screen, we found that only 5 compounds elicited a differential effect on the viability of *Pkd1-*null vs. wt cells, while the majority had effects independent of the *Pkd1* status. We assayed these 5 compounds (the mTOR inhibitor Everolimus, the Chk1 inhibitor AZD-7762, the Ikk inhibitor IMD-0354 and the Plk1 inhibitors Volasertib and Rigosertib) in our Matrigel-based 3D assay and found that all inhibited cyst growth (Supplementary Table 5).

### Phenotypic screening of a panel of ADPKD and normal human kidney (NHK) epithelial cells

**Figure 5:**
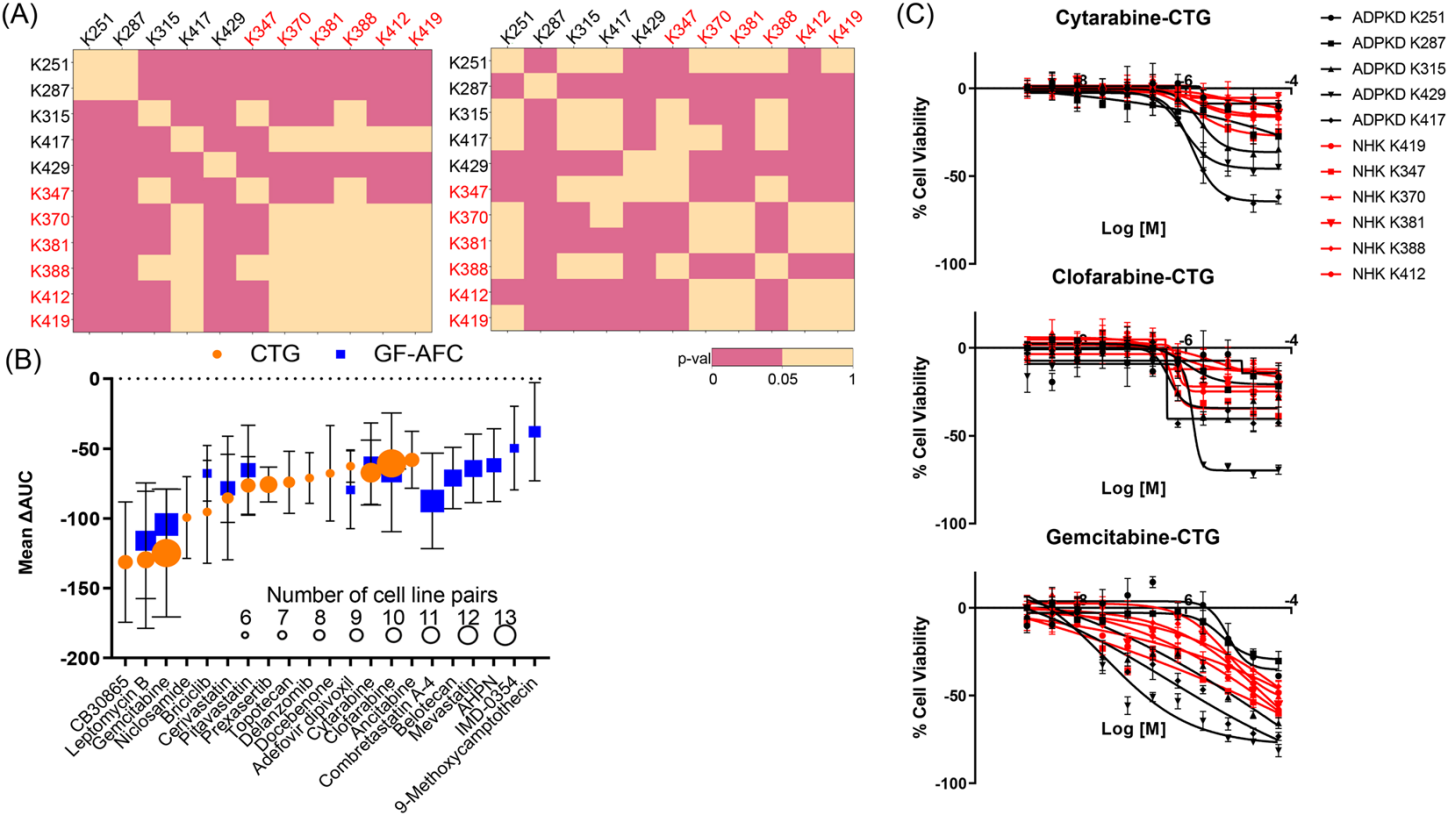
Compound responses in a panel of primary ADPKD and normal human kidney (NHK) epithelial cells. (**A**) Pairwise analysis of similarity of compound response between kidney cell preparations. Cell pairs displaying similar responses (p-val > 0.05) are colored yellow and those displaying different responses (p-val < 0.05) are colored pink. ADPKD cells are labeled in black and NHK in red. Left: CTG readout; Right: GF-AFC readout. (**B**) Mean ΔAUC ± SD of compounds with differential response in ADPKD vs. NHK cell pairs. Only compounds with differential responses in more than 6 pairs (with at least 2 different ADPKD and 3 different NHK cell isolates) are shown. The size of the node corresponds to the number of cell pairs. (**C**) Dose response curves of antimetabolites displaying differential response in pairwise analysis of ADPKD (black) and NHK (red) primary cells in the CTG readout. Responses obtained with GF-AFC reagent are shown in Supplementary Fig. 9. Data is represented as Mean ± SD, n = 3.

In order to identify effective compounds and therapeutic targets relevant to human disease we obtained a panel of primary human cells from the PKD Biomarkers and Biomaterials Core at the University of Kansas Medical Center. The panel consisted of 5 ADPKD cystic (K251, K287, K315, K417 and K429) and 6 NHK cell cultures (K347, K370, K381, K388, K412 and K419). We screened each cell preparation against the collection of 109 hits identified above. We also included 46 compounds that were inactive in the cyst growth assay to validate the triaging strategy. Similar to the screen of murine cells, compounds were tested using a dose response curve in a 1,536-well format, and the viability of human primary cells was measured after 48 hours of compound treatment using GF-AFC and CTG readouts (assay statistics provided in Supplementary Table 1). We first performed a pairwise analysis to determine how similar is the response to compound treatment among ADPKD and NHK cell preparations. In the CTG readout, we observed that overall NHK cells showed similar compound responses among each other and different to that of ADPKD cells. In contrast, compound responses in the ADPKD cells were more variable (Fig. 5A, left). Interestingly, the response of certain ADPKD cell preparations (i.e. K417) to the compound set was more similar to that of NHK cells than to other ADPKD types. The similarity pattern was less obvious when assessing compound responses based on the GF-AFC readout (Fig. 5A, right). This indicates that not all disease and normal cells behave the same when exposed to the set of 155 hit compounds. The underlaying genotypic/phenotypic background differences could explain this observation. In addition, cells obtained from ADPKD patients were isolated from multiple cysts of unknown origin. While most of larger cysts are thought to be derived from collecting ducts, the overall contribution of cystic cells from other segments of the nephron remain unknown and may contribute to the variability in the response to compounds targeting specific pathways.

To identify compounds with robust bioactivity profiles against some human ADPKD samples but minimal or less pronounced effects in most NHK cells, we performed a pairwise analysis of compound response using ΔAUC values in both GF-AFC and CTG readouts (see Materials and Methods for activity cutoffs). We then identified 21 compounds that displayed a differential response in at least 6 out of the 30 total pairs of ADPKD-NHK cells (the 6 minimum pairs contained at least 2 ADPKD and 3 NHK different cell types) (Fig. 5B). Except for Niclosamide, Docebenone and Mevastatin, the remaining 18 compounds identified in Fig. 5B also showed an effect in the cyst growth assay (Supplementary Table 5), indicating that our triaging strategy identified relevant compounds. The antimetabolites Cytarabine, Clofarabine and Gemcitabine were the compounds that showed differential responses in the most pairs of ADPKD and NHK cells (Fig. 5C and Supplementary Fig. 9). Interestingly, Leptomycin B is the only compound from the initial reference set that showed a differential response in most pairs of ADPKD but not NHK cells (Fig. 5B).

## Discussion

Here we present a high-throughput screening platform to identify cancer drugs that could be potentially repurposed for the treatment of ADPKD. First, our platform implements a titration-based screen to identify compounds that preferentially affect the viability/proliferation of *Pkd1-*null cells but have minimal effect on wt cells. Because the screen utilizes cells of murine origin grown in monolayers and is fully automated, it allows the assessment of a large number of compounds. From a screen of 8,814 unique compounds with oncological annotations, we identified 155 compounds with differential effects on *Pkd1-*null vs. wt cells. These compounds target multiple pathways already implicated in ADPKD and related disorders, which highlights the validity of the screen. Additionally, we identified putative therapeutic classes not previously associated to ADPKD such as DNA Topoisomerase and Sphingosine Kinase (SphK) inhibitors, of which the first class also reduced *in vitro* cyst growth. As the readout of the primary screen is cell viability/proliferation, our platform will miss therapeutically relevant compounds that do not lead to quantifiable differences in cell numbers between mouse *Pkd1-*null and wt cells, at least during the time frame of our assays. Contrary to *in vivo* models, the effect of Tolvaptan and CFTR inhibitors on *in vitro* cell proliferation requires the addition of cAMP-stimulating agents^22,26^. Since the assays in this study were performed in the absence of cAMP-stimulating agents, the compounds identified likely function through pathways unrelated to cAMP. The use of cAMP stimulators in future high-throughput screens, would likely facilitate the identification of compounds that reduce cyst formation/growth through modulation of cAMP levels. Second, our platform incorporates a more relevant secondary 3D assay to evaluate the effect of compounds on cyst growth. Among the 155 compounds identified in the large-scale screen, this secondary 3D assay validated 109 of them. From a therapeutic point of view, molecules that affect the proliferation or viability of cells might not be desirable for prolonged use, at least in patients at early disease stages. However, agents that reduce cyst swelling without major effects on cell viability or growth may be more suitable for this purpose. Importantly, we identified numerous compounds that visibly reduce cyst swelling without major effects on the viability readout. Among these, we found Paclitaxel, Epothilone A and D, and several antiparasitics, all of which affect tubulin dynamics. Indeed, the microtubule cytoskeleton has been implicated in the pathogenesis of PKD^9^. Another two compounds that reduced cyst swelling without major effects on viability were the ROCK 1/2 inhibitor GSK-269962A and the DNMT1 inhibitor 5-azacytidine. In support of our findings, it has been recently reported that ROCK inhibitors and DNA methylation inhibitors decrease cyst formation in 3D cultures of *PKD1* mutant mIMCD3 cells and canine kidney cells, respectively^42,43^. Lastly, Tosedostat, an inhibitor of the M1 family of aminopeptidases, also reduced cyst formation without affecting viability. However, to the best of our knowledge, a role of M1 aminopeptidase inhibition in cyst reduction has not been reported. Altogether, these compounds merit further investigation as potential treatments for ADPKD. Alternative therapeutic strategies designed to prevent or eliminate cyst formation early on, as opposed to reducing the growth of already formed cysts, have been proposed^44^. In the future, it will be interesting to test the set of compounds identified here for their effect on cyst formation in a cystogenic 3D assay, where cells are treated with compounds before any cyst is visibly formed.

Several therapies have shown promise in animal models of ADPKD but have not been translatable to human disease^36,45^. To address this issue, our pipeline incorporates the evaluation of compound efficacy in human primary cells. Because these patient-derived cells cannot be expanded to sustain the screen of large compound collections, we limited our screen to those compounds that previously showed an effect on mouse cells, while realizing that compounds with no effect on mouse cell models but with potential effects on human cells might have been missed. Notably, we observed differences in compound responses amongst ADPKD and normal cell isolates, suggesting that patient genotypic/phenotypic background differences could have a significant impact on treatment outcomes. Additionally, most human cell isolates come from several cysts (distal and proximal tubules) pooled and digested together, which further contributes to the observed variability. It will be interesting to determine whether this variability may represent the potential responses to specific drugs given to patients. Nevertheless, a high-throughput screen would benefit from a better cell model that is clonal in nature and immortalized to allow multiple passages and expansions to obtain large cell numbers. Future generation of paired human normal and *PKD1-null* renal epithelial tubule cell lines of clonal nature and immortalized would be a great tool to facilitate high-throughput screening and drug repurposing for ADPKD.

We were able to identify 21 compounds that preferentially affected the viability of most ADPKD vs. normal kidney cell isolates. The effect of some of these compounds and underlying mechanisms (Niclosamide, Briciclib, Prexasertib, AHPN, and IMD-0354) have not been studied in the context of ADPKD. Compounds such as Cytarabine and its prodrug Ancitabine, Clofarabine, Gemcitabine, Adefovir, and the DNA Topoisomerase inhibitors Topotecan, Belotecan, and 9-methoxycamptothecin, likely limit the proliferation of ADPKD cells by directly interfering with DNA replication. In contrast, other compounds and/or mechanisms have been previously implicated in the pathogenesis of ADPKD and constitute good candidates for follow up studies. Among these are compounds that affect microtubule dynamics (Combretastatin A-4), inhibitors of nuclear transport (Leptomycin B), proteasome inhibitors (Delanzomib), inhibitors of NAD synthesis (CB30865), which others have also proposed as potential therapeutic options for ADPKD^17,38,46^. Additionally, the identification of HMG-CoA reductase inhibitors or “statins” (Cerivastatin, Pitavastatin, and Mevastatin) as potential therapeutic options is supported by studies not only in rodent models but also in ADPKD patients where statins diminished the severity of the disease^47,48^. Many of the above mentioned compounds are either approved or have been tested in clinical trials for other ailments and would be interesting candidates for repurposing in ADPKD, albeit for short term treatment or in patients with advanced disease. In the future, it will be of interest to investigate the effect of these compounds in combination studies.

In summary, the platform presented in this study is a viable avenue to identify and/or repurpose chemical agents for ADPKD. To the best of our knowledge, this is the first high-throughput study aiming at repurposing drugs for ADPKD. We hope the compounds identified here will serve the scientific community as starting points for future efficacy studies utilizing relevant *ex vivo* and *in vivo* ADPKD models.

## Methods

### Compound libraries

The NIH Chemical Genomics Center (NCGC) Pharmaceutical Collection (NPC) contains 2,816 compounds of which 50% are approved by the United States Food and Drug Administration (FDA). The remaining drugs are in use in other countries and/or have been tested in clinical trials against a range of diseases^11^. The NCATS Pharmacologically Active Chemical Toolbox (NPACT) library contains 5,099 highly annotated compounds that target most known biological mechanisms and pathways (manuscript in preparation). Many of the known mechanisms are represented by a few best-in-class compounds with non-redundant chemotypes that provide a diversity of physicochemical and pharmacological properties. Chemical classes include synthetic small molecules as well as microbial- and plant-derived purified natural products (https://ncats.nih.gov/preclinical/core/compound/npact). The Kinase Inhibitor collection contains 977 clinical and pre-clinical-stage compounds that inhibit one or more kinases^33^. The MIPE (Mechanism Interrogation Plate) collection contains approved and investigational 2,480 oncology-focused agents^30–32^.

### Cell lines and culture conditions

Mouse postnatal PH2 and PN24 cells were of proximal tubule origin (provided by S. Somlo through the George M. O’Brien Kidney Center, Yale Univ., New Haven, CT) and generated and cultured as described^39,49,50^. MEK-wt and MEK-null cells were of collecting duct origin (provided by X. Li through the Jared Grantham Kidney Institute, Univ. Kansas Medical Center, Kansas City, KS) and generated and cultured as previously described^29,51^. Cell identity was validated as described^52^. Briefly, MEKs were grown in DMEM/F12 (ThermoFisher) supplemented with 2% FBS (Hyclone), 2.5 mM glutamine (ThermoFisher), 0.6 ng/mL mouse interferon-γ (Sigma), 1x Insulin-Transferrin-Selenium (ThermoFisher), 100 nM 2,3,5-triido-L-thyronine (Sigma), 36 ng/mL hydrocortisone (Sigma), and 1,000 U/mL penicillin-streptomycin (ThermoFisher). PN24 and PH2 cells were grown in DMEM/F12, supplemented with 2.5 mM glutamine, 10% FBS, 1 ng/mL interferon-γ and penicillin-streptomycin as above. Human primary normal and ADPKD renal epithelial cells were obtained from the PKD Biomarkers and Biomaterials Core at Kansas University Medical Center and cultured as described^26^. Briefly, cells were cultured in DMEM/F12 supplemented with 5% FBS, 1x Insulin-Transferrin-Selenium and 1,000 U/mL penicillin-streptomycin. Cells were maintained in a 37 °C incubator with 5% CO_2_ and under a humidified atmosphere and routinely tested for mycoplasma contamination using MycoAlert mycoplasma detection kit (Lonza). Cells were passaged no more than 3 times before using.

### qHTS cell viability assay

Assays were performed as described before^53^ with some modifications. Cells were assayed in growth media at a density of ~800 cells/well. Four μL of cells were dispensed into 1,536-well, white, solid-bottom, TC-treated plates (Greiner Bio One) using a Multidrop dispenser and incubated at 37 °C, 5% CO_2_, under a humidified atmosphere for 5 hours. Twenty-three nL of compounds and controls (neutral control DMSO or positive control Digitonin at final concentration of 114 µM) were subsequently transferred via Kalypsys Pin-tool. For primary screens, Kinase Inhibitor and NPACT collections were tested at 7 concentration points ranging from 0.36 nM to 57 µM, and NPC compounds were tested at 8 concentration points ranging from 0.73 nM to 57 µM. MIPE agents were tested at 11 concentration points ranging from 0.96 nM to 57 µM. Validation screens and screens of human cell isolates were performed in triplicate at 11-point dilutions using fresh compound dilutions (final concentration range of 0.96 nM to 57 µM). Cells were incubated for 48 hours followed by viability measurements: First, 1 μL of 5x GF-AFC (MP Biomedical; 125 µM in 10 mM HEPES, pH 7.5 to achieve a final concentration of 25 µM) was added to each well and plates were incubated 30 minutes at 37 °C and analyzed for fluorescent intensity using a ViewLux High-throughput CCD imager equipped with Ex405/10 and Em525/20 FITC filters. Second, 3 µL of CellTiter-Glo (Promega) were subsequently added to each well and plates were incubated for 15 min at RT and analyzed for luminescence intensity using a ViewLux High-throughput CCD imager equipped with clear filters.

### 3D cyst assay

Cells were cultured in growth media without interferon-γ for 7 days prior to harvesting. Ten μL of cells/well (at a density of 5,000 cells/well) were dispensed into 384-well, black, flat, clear-bottom, ULA plates (Corning) using a Multidrop dispenser and plates were placed on ice. Forty μL/well of a cold 50% Matrigel solution (in growth media) were immediately added using multichannel pipette and cells were mixed with Matrigel solution by gentle pipetting to avoid formation of bubbles (final Matrigel concentration of 40%). Plates were centrifuged at 1,000 rpm for 1 min, sealed with breathable seals and incubated at 37 °C, 5% CO_2_, under a humidified atmosphere for 4 days. Fifty μL/well of a solution of compounds and controls (neutral control DMSO or positive control Leptomycin B at final concentration of 10 µM; final 1% DMSO in all cases) in media were subsequently transferred using a multichannel pipette. Compounds were tested at 8 concentration points ranging from 100 to 0.78 µM in triplicate. Compounds that were identified to reduce cyst growth even at the lowest concentration tested, were re-tested at a concentration range of either 50 to 0.39 or 10 to 0.078 µM. Plates were sealed with breathable seals and incubated at 37 °C, 5% CO_2_ for 5 additional days. Plates were imaged using an Opera Phenix high-content screening system (PerkinElmer) in confocal mode (brightfield, 5x air objective, 30 Z sections at 25 µm per section). The image analysis was done at each stack with the Columbus Image Analysis System (PerkinElmer) using the build-in analysis function. The cyst region was identified using bright-field at cutoff 3500 µm^2^ of cyst area and threshold 0.4 region signal to background ratio. The cysts were further characterized with different texture selection. In order to locate the cysts in the most focused z stack for every well, the focused plane was then further filtered with a customized MatLab script based on the bright-field SER ridge texture. To assess cell viability, media was removed by inverting over a plate adaptor to collect media and gently tapping to prevent disruption of 3D gel. Fifty µL/well of CTG-3D (Promega) were subsequently added and plates were shaken at 500 rpm for 30 min at RT and analyzed for luminescence intensity using a ViewLux High-throughput CCD imager equipped with clear filters. The Supplementary Movie was obtained using the IncuCyte S3Live-Cell analysis system (Essen Bioscience). Cyst staining showed on Fig. 3B was performed by removing media from wells on the last day and adding 50 µL of a 1:200 dilution of WGA-Alexa 488 (5 µg/mL stock; ThermoFisher) and 1:2000 dilution of Hoechst (ThermoFisher) in PBS. The solution was incubated overnight at 4 °C and wells were washed with PBS twice before imaging in the Opera Phenix.

### qHTS data analysis and statistics

The screening data were analyzed using software developed internally at the NIH Chemical Genomics Center. Data from each assay were normalized plate-wise to corresponding intra-plate controls as described previously^54^. The same controls were also used for the calculation of the Z’ factor index for each assay^55^. Supplementary Table 1 shows S:B and Z’ values for all primary and validation screens. Percent activity was derived and fitted to the Hill equation using in‐house software^56^. Concentration-response curves (CRCs) were classified as described previously and compounds exhibiting high-quality CRCs (class −1 and −2) were considered active^13^. To identify compounds with differential response in *Pkd1*-null vs. wt cells in primary and validation screens in monolayer of mouse MEK and PN cells, the ΔAUC (AUC_Pkd_ − AUC_wt_) for each readout (CTG or GF-AFC) was calculated and those compounds exhibiting high-quality CRCs (−1 and −2) for *Pkd1*-null cells and ΔAUC < −10 or −20 for primary and validation screens, respectively, were considered as having differential activity in *Pkd*-null vs. wt cells (Supplementary Tables 2 and 3). Venn diagrams presented on Fig. 2 were generated using Venny software^57^.

To identify compounds that reduce cyst growth of PN24 cells grown in a 3D gel, the CTG viability readout was used to determine activity cutoff given its superior assay performance compared to imaging-based readouts. All compounds with a curve class of −1, −2 and 5 (non-class 4) and AUC < −100 were considered active. Visual inspection of curves was also performed to ensure adequate classification of compounds.

To identify compounds with differential responses in monolayer screens of ADPKD vs. NHK cells, the differential response of each compound was calculated for each of the 30 pairs of ADPKD and NHK cells (5 ADPKD and 6 NHK isolates) in both the CTG and GF-AFC readouts. Compounds with high-quality CRCs (classes −1 and −2) for ADPKD cells, showing an ΔAUC < −60 and Δ|Zero activity| < 10 with respect to NHK curves were considered to show a differential response. Visual inspection of curves was also performed to ensure adequate classification of compounds. Compounds showing differential activity in at least 6 pairs of ADPKD vs. NHK cells (pairs consisted of at least 2 different ADPKD and 3 different NHK cell types) were highlighted in Fig. 5B.

### Target enrichment analysis

To perform the target enrichment analysis in Fig. 2C we utilized multiple statistical comparisons of the occurrences of primary gene targets of compounds for two sets of compounds calculating adjusted probabilities using a Benjamini-Hochberg correction. This method reduces the false positive outcomes due to incorrect target identification. Thus, statistical analysis of two gene target sets were performed. The first set includes the gene symbols of primary targets of the active compounds, while the second set includes the gene symbols of primary targets of all compounds tested. Only genes with adjusted p-value more than 0.05 in the first set were considered as statistically significantly different from the second set.

### Pairwise analysis of compound response similarity

To compare general effects of selected compounds across the ADPKD and NHK cells (5 ADPKD and 6 NHK isolates), we performed a pairwise analysis of compound AUC values in both GF-AFC and CTG readouts. Paired samples Wilcoxon test^58^ was used to calculate population differences of the compounds AUC values for each pair of isolates. A full comparison matrix of the 11 isolates with probability values was constructed and coverted to a heat map. Statistical analysis was performed using KNIME analytic platform^59^ and heat map visualization was implemented using Matplotlib library^60^.

## Supplementary information

Supplementary information.

Supplementary movie.

Supplementary table 1.

Supplementary table 2.

Supplementary table 3.

Supplementary table 4.

Supplementary table 5.

## Data Availability

The datasets generated in the current study are available in Supplementary Information and from the corresponding author on reasonable request.
