# Author Correction: A high-throughput screening platform for Polycystic Kidney Disease (PKD) drug repurposing utilizing murine and human ADPKD cells

**DOI:** 10.1038/s41598-022-21947-1

**Published:** 2022-10-13

**Authors:** Rosita R. Asawa, Carina Danchik, Alexey Zakharov, Yuchi Chen, Ty Voss, Ajit Jadhav, Darren P. Wallace, Josephine F. Trott, Robert H. Weiss, Anton Simeonov, Natalia J. Martinez

**Affiliations:** 1 National Center for Advancing Translational Sciences, National Institutes of Health, Rockville, MD USA; 2 Department of Internal Medicine, University of Kansas Medical Center, Kansas City, KS USA; 3 Division of Nephrology, Department of Internal Medicine, University of California, Davis, CA USA

Correction to: *Scientific Reports* 10.1038/s41598-020-61082-3, published online 06 March 2020

The original version of this Article contained an error in the spelling of the author Alexey Zakharov which was incorrectly given as Alexey Zahkarov. The original Article and accompanying Supplementary Information 1 file have been corrected.

