# Supplementary material for: A high-throughput screening platform for Polycystic Kidney Disease (PKD) drug repurposing utilizing murine and human ADPKD cells
Source: Sci Rep. 2020 Mar 6;10:4203. doi: 10.1038/s41598-020-61082-3 (PMC7060218; doi:10.1038/s41598-020-61082-3)

SUPPLEMENTARY INFORMATION

A high-throughput screening platform for Polycystic Kidney Disease (PKD) drug repurposing utilizing murine and human ADPKD cells

Rosita R. Asawa[1], Carina Danchik[1], Alexey Zakharov[1], Yuchi Chen[1], Ty Voss[1], Ajit Jadhav[1], Darren P. Wallace[2], Josephine F. Trott[3], Robert H. Weiss[3], Anton Simeonov[1], and Natalia J. Martinez[1*]

[1]National Center for Advancing Translational Sciences, National Institutes of Health, Rockville, MD, USA

[2]Department of Internal Medicine, University of Kansas Medical Center, Kansas City, KS, USA

[3]Division of Nephrology, Department of Internal Medicine, University of California, Davis, CA, USA

*Corresponding author (natalia.martinez@nih.gov)

The Supplementary Information file contains figures and legends for Supplementary Figures 1 through 9. It also contains the legends of Supplementary Tables 1-5, which are provided in associated excel files.

Supplementary Figure 1. Growth rate of mouse cells during time course of assay. For each cell type, viability was determined using GF-AFC (left) and Cell Titer-Glo (CTG, right) reagents at 0, 24 and 48 hr after plating into 1,536-well plates. Growth rate is represented as percent signal normalized to 0 hr (0%) and 48 hr (100%).

Supplementary Figure 2. **Cell viability outcome of reference set compounds.** Dose-response curves of the reference set compounds that showed differences in the AUC between at least one pair of *Pkd1*-null

(black) and wt cells (red) as exemplified in Figure 1A. Data is represented as Mean ± SD, n=3. Unpaired, parametric (mean AUC) T-test (Welch's correction) *p-val <0.05; **p-val<0.001; ***p-val<0.0001.

Supplementary Figure 3. Cyst-growth assay. (A) Bright-field image at 5x magnification of PN24 cysts (top) and MEK-null cysts (bottom) grown in 40% Matrigel for 3 (left) or 5 (right) days. Scales bars are shown in red. (B) Confocal imaging of cysts show lumen swelling. Cysts grown for 9 days were stained with Hoechst and imaged at 10x magnification using confocal microscopy. Fluorescent (top) and bright-field merged (bottom) images of 6 Z planes (25 µm apart) are shown. A schematic of each plane is shown on the bottom. Scales bars are shown in red. (C) Left: Representative image of a well containing cysts and analyzed using the Columbus Image Analysis System (PerkinElmer). The cyst region was identified in the bright-field at cutoff of 3500 μm^2 of cyst area and 0.4 regional signal to background ratio. Cyst in the margin of the well were removed from analysis. Green indicates cysts that pass the cutoff while red indicates cysts that did not meet the cutoff. Right: Plot of cyst are of all objects identified in the well. Dotted line indicates cutoff.

Supplementary Figure 4. Optimization of the 3D cyst growth assay. Cells were seeded at 100, 500, 1000, 2000 or 5000 cells per well of a 384-well plate in 40% Matrigel and allowed to form cysts for a total of 9 days. Representative images are shown in (A). (B) Cyst number/well (left), total cyst area/well ([μm^2], middle) and average cyst area/well ([μm^2], right); all graphs show average and standard deviation of n=16. (C) Bright-field image at 5x magnification of PN24 cysts grown in 40% Matrigel and treated for 9 days with different DMSO concentrations as indicated. A concentration of 1% was chosen for subsequent experiments.

Supplementary Figure 5. Reference set compounds reduce cell aggregates and/or cyst swelling in 3D culture. (A) Representative images of cysts grown in 40% Matrigel for 4 days and treated with the indicated compounds for 5 additional days. Each compound was tested at a total of 8 concentration points (1:2 serial dilutions). Everolimus, Tracolimus, and KPT-9274 were tested at a concentration range of 100 to 0.78 µM;

KPT-330, Emodin, and Paclitaxel at 50 to 0.39 µM; Triptolide, Quisinostat, KPT-335, and Leptomycin B at 10 to 0.078 µM. (B) Graphs indicate dose response curves of each compound in the CTG (grey circles) and imaging-based cyst size (purple squares) readouts. Data is represented as Mean ± SD, n=3.

Supplementary Figure 6. Compounds from the reference set that do not elicit differential viability outcomes between *Pkd1-null* and wt cells in the 2D assay but reduce growth of PN24 cysts. Graphs on the left indicate dose response curves of each compound in the CTG (grey circles) and imaging-based cyst size (purple squares) readouts. Data is represented as Mean ± SD, n=3. Representative images of cysts grown in 40% Matrigel for 4 days and treated with the indicated compounds for 5 additional days are shown on the right. Each compound was tested at a total of 8 concentration points (1:2 serial dilutions), ranging from 100 to 0.78 µM.

Supplementary Figure 7. Compounds that affect tubulin dynamics reduce cyst swelling. Graphs on the left indicate dose response curves of each compound in the CTG (grey circles) and imaging-based cyst size (purple squares) readouts. Data is represented as Mean ± SD, n=3. Representative images of cysts grown in 40% Matrigel for 4 days and treated with either the indicated compounds for 5 additional days are shown on the right. Each compound was tested at a total of 8 concentration points (1:2 serial dilutions). Flubendazole, Albendazole, and Oxibendazole were tested at a concentration range of at 50 to 0.39 µM; Epothilone D was tested at 100 to 0.78 µM.

Supplementary Figure 8. Additional compounds that reduce cyst swelling and have minimal effect on viability. Graphs on the left indicate dose response curves of each compound in the CTG (grey circles) and imaging-based cyst size (purple squares) readouts. Data is represented as Mean ± SD, n=3. Representative images of wells treated with the indicated compound are shown on the right. Each compound was tested at a total of 8 concentration points (1:2 serial dilutions). Both, Azacytidine and GSK-269962A were tested at a concentration range of 50-0.39 µM.

Supplementary Figure 9. Dose response curves of antimetabolites displaying differential response in pairwise analysis of ADPKD (black) and NHK (red) primary cells in the GF-AFC readout. Data is represented as Mean ± SD, n=3.

Supplementary Table 1: Plate-based assay statistics. Mean ±SD Signal-to-Background (S:B) and Z' are shown for each assay. Readouts of viability are GlyPhe-7-Amino-4-Trifluoromethylcoumarin (GF-AFC) and CellTiter-Glo (CTG) for primary and validation assays in monolayer cultures. For the 3D cyst growth assay the readouts are CTG and cyst size (μm^2) (determined by imaging-based analysis). Primary screens n=62-88 plates; validation screen in monolayer n=6 plates; cyst assay n=12.

Supplementary Table 2: Compounds identified from primary screens displaying a differential response in *Pkd1*-null vs. wt cells. Compound exhibited high-quality CRCs (either -1 or -2) for *Pkd1*-null cells and $\Delta AUC_{\mathit{Pkd1}\text{null-wt}}$< -10. Note for certain compounds, multiple sample IDs were found. NA: non-applicable.

Supplementary Table 3: Compounds identified from validation screens displaying a differential response in *Pkd1*-null vs. wt cells. Compound exhibited high-quality CRCs (either -1 or -2) for *Pkd1*-null cells and $\Delta AUC_{\mathit{Pkd1}\text{null-wt}}$< -10. Note for certain compounds, multiple sample IDs were tested.

Supplementary Table 4: Primary mode of action (MOA) and indication of compounds identified in validation screens that elicit a differential response in *Pkd1*-null vs. wt cells.

Supplementary Table 5: Activity of compounds tested in the 3D cyst growth assay. Compounds that exhibited curve classes (CC) of -1, -2, -3 or 5 classes (non-class 4) and an AUC< -100 in the CTG readout were considered active. Visual inspections of curves were also performed to ensure proper compound classification and although some compounds did not meet one of the cutoffs (CC or AUC) they were considered active.

Supplementary Figure 1

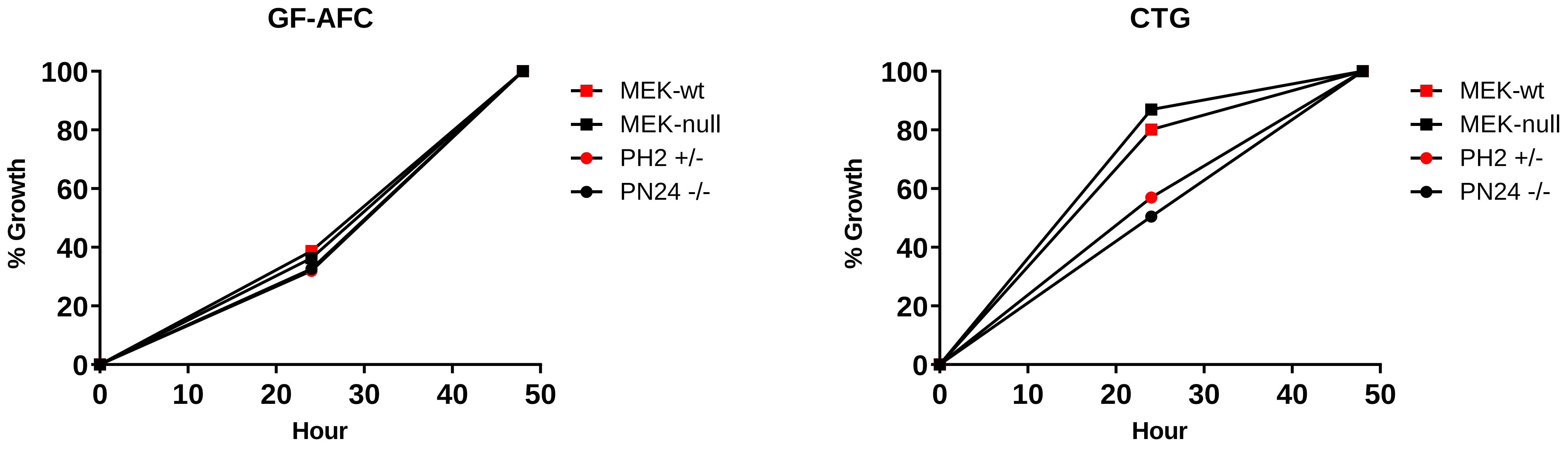

Supplementary Figure 2

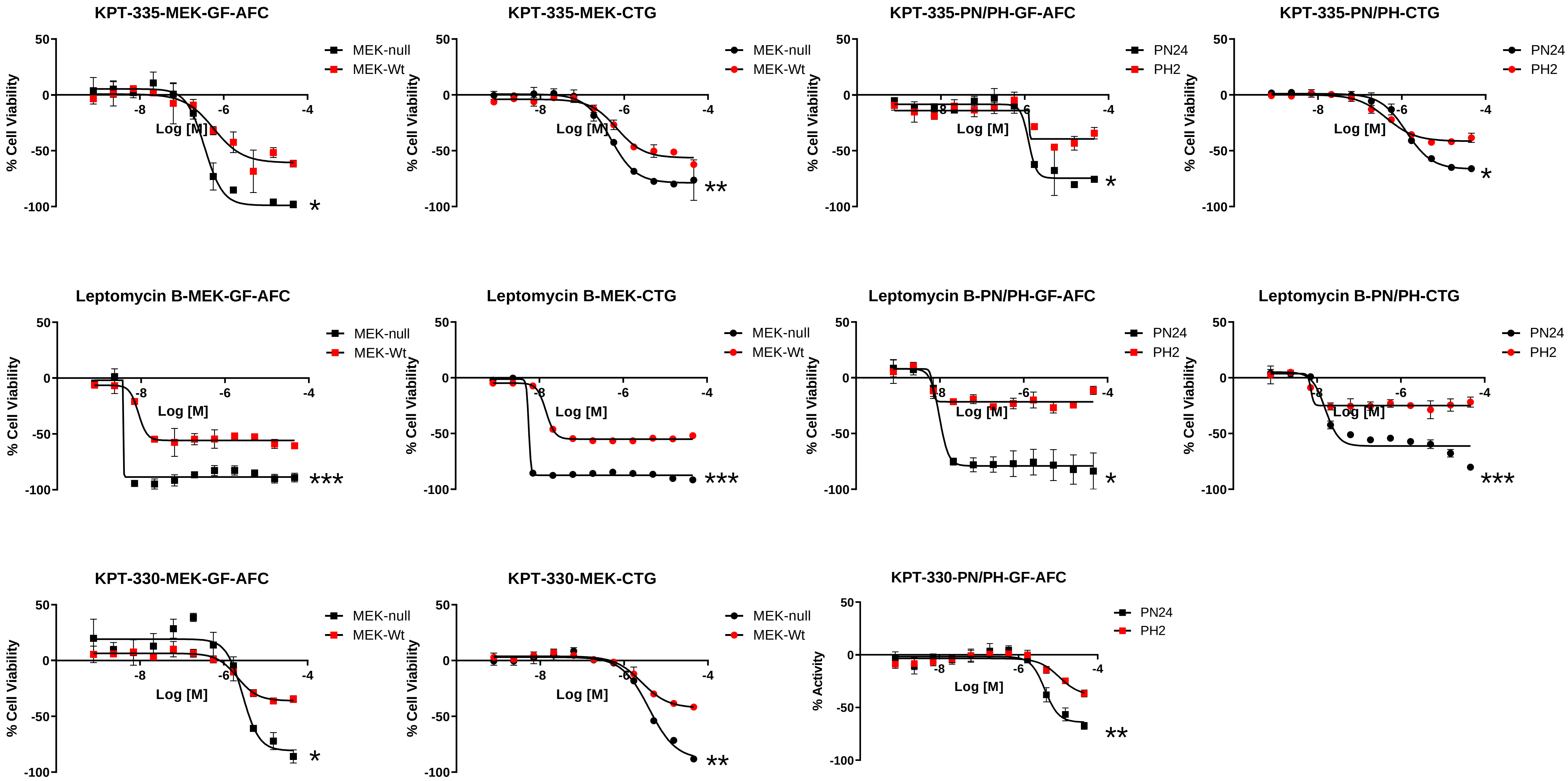

Supplementary Figure 2 cont.

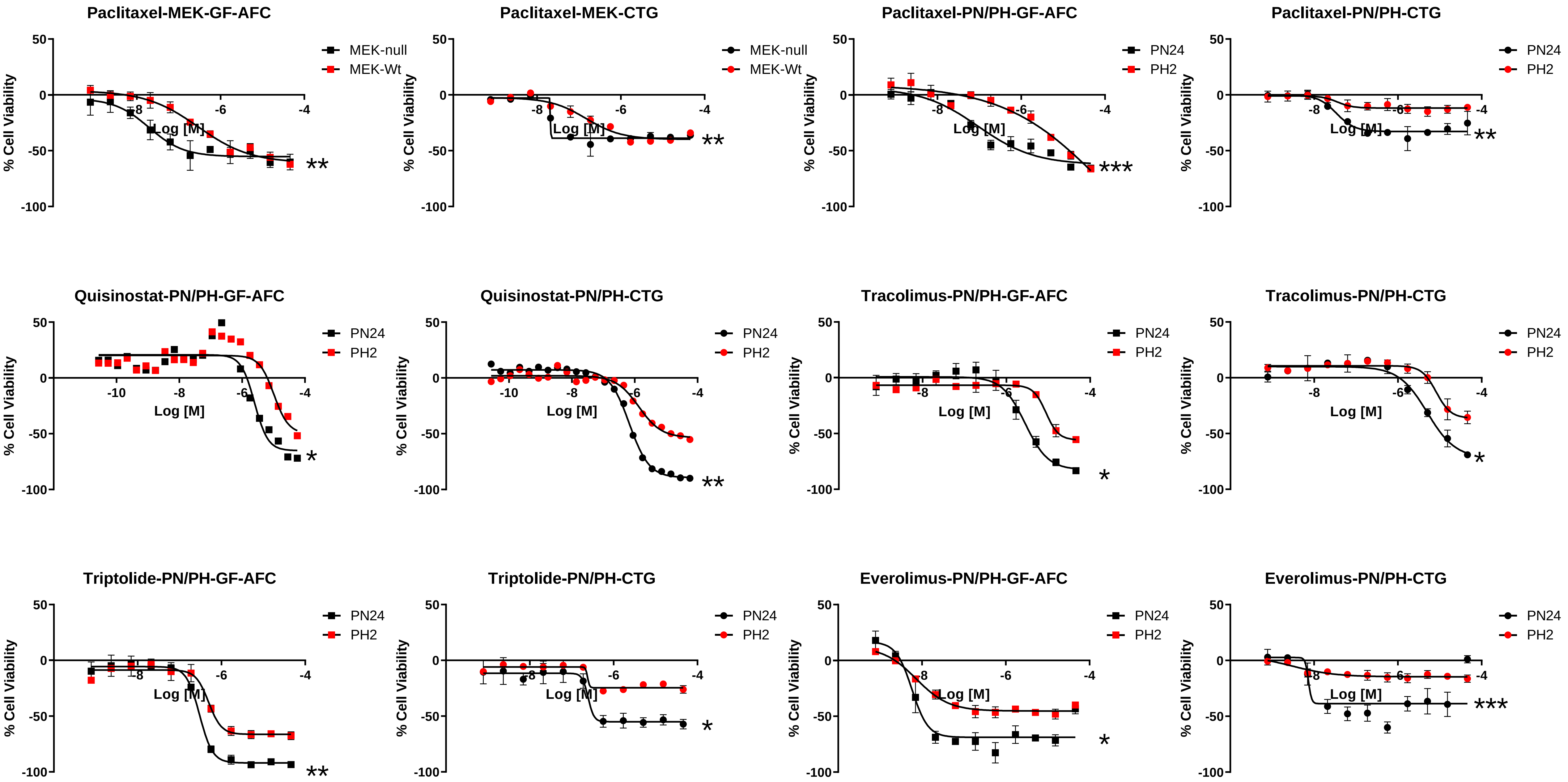

Supplementary Figure 2 cont.

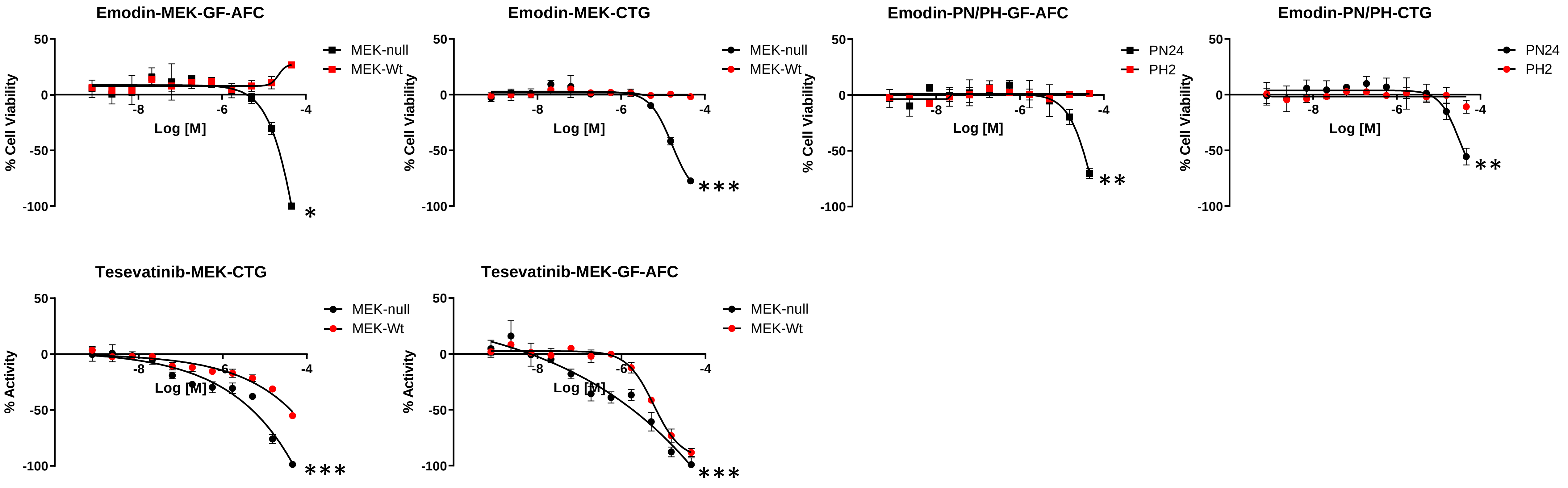

Supplementary Figure 3

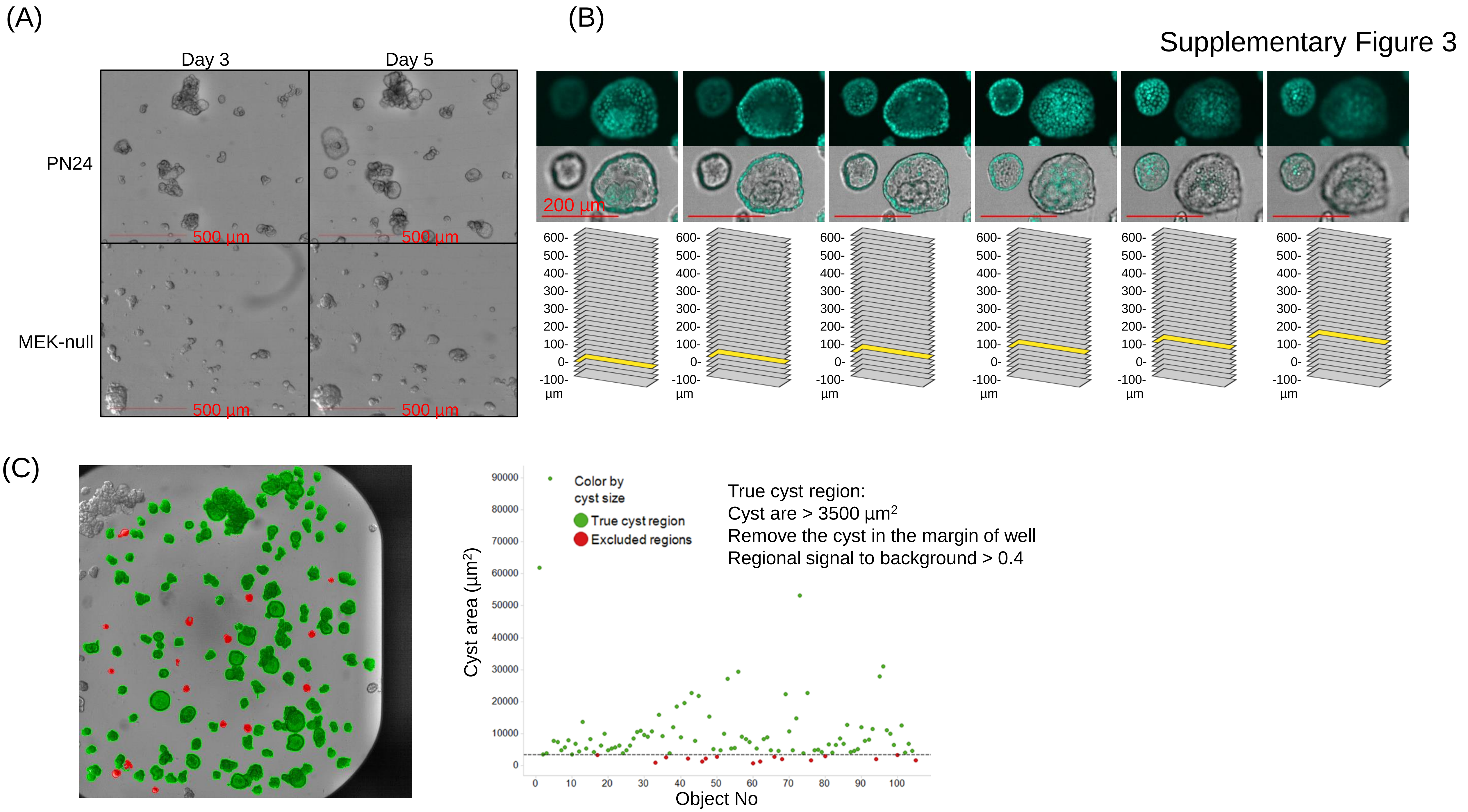

Supplementary Figure 4

(A)

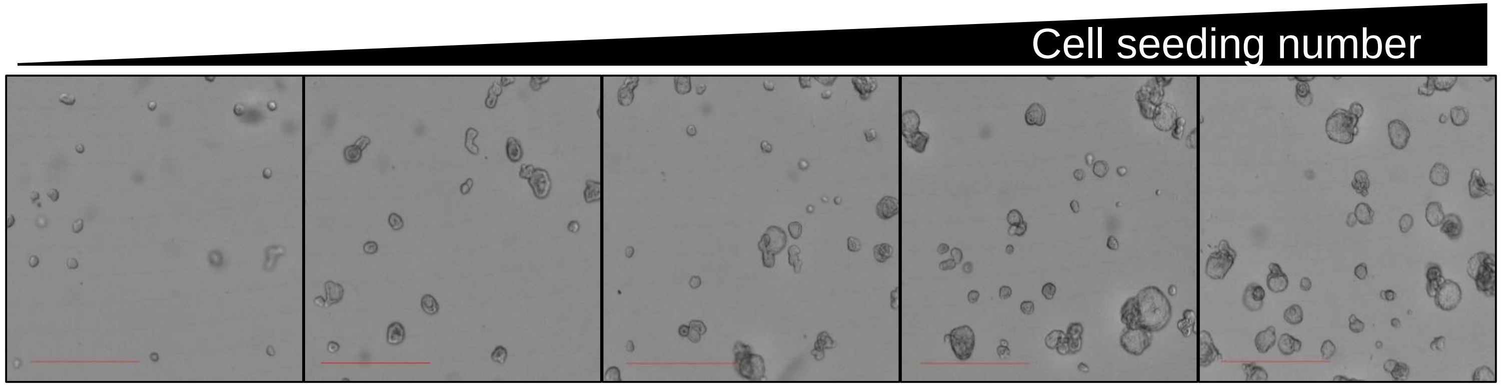

(B)

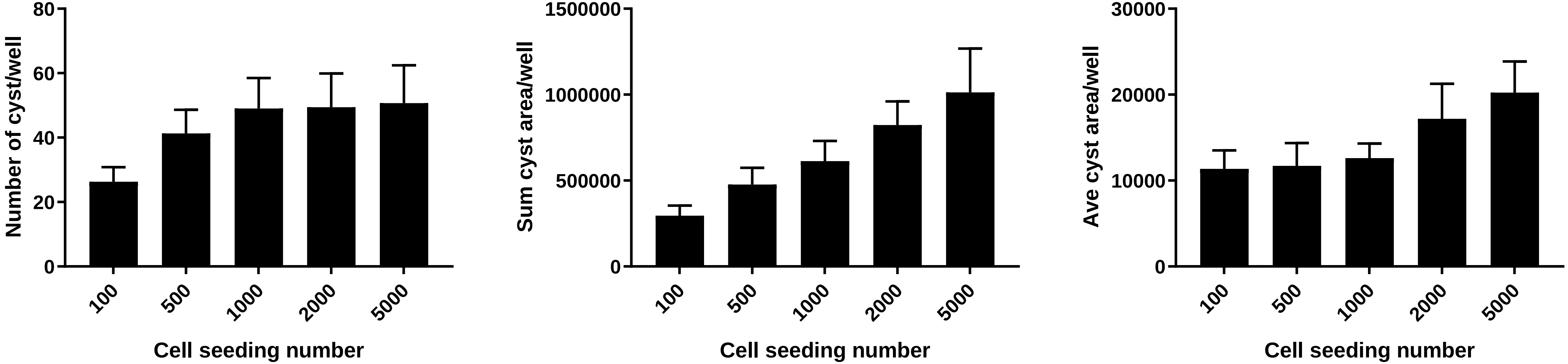

(C)

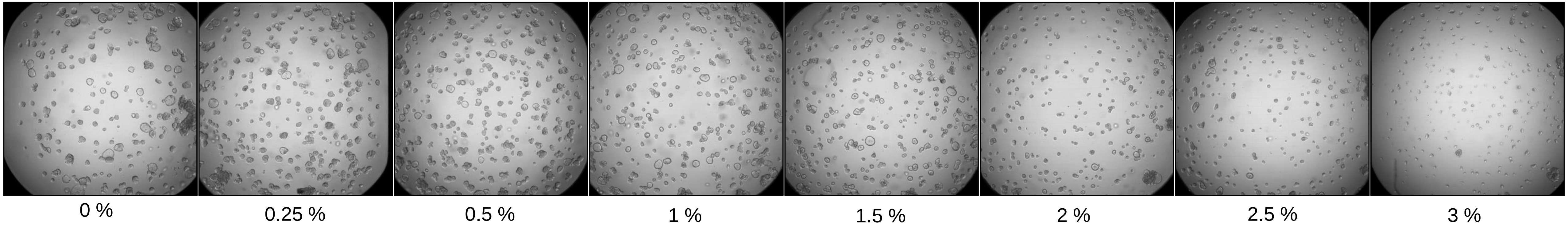

Supplementary Figure 5 (A)

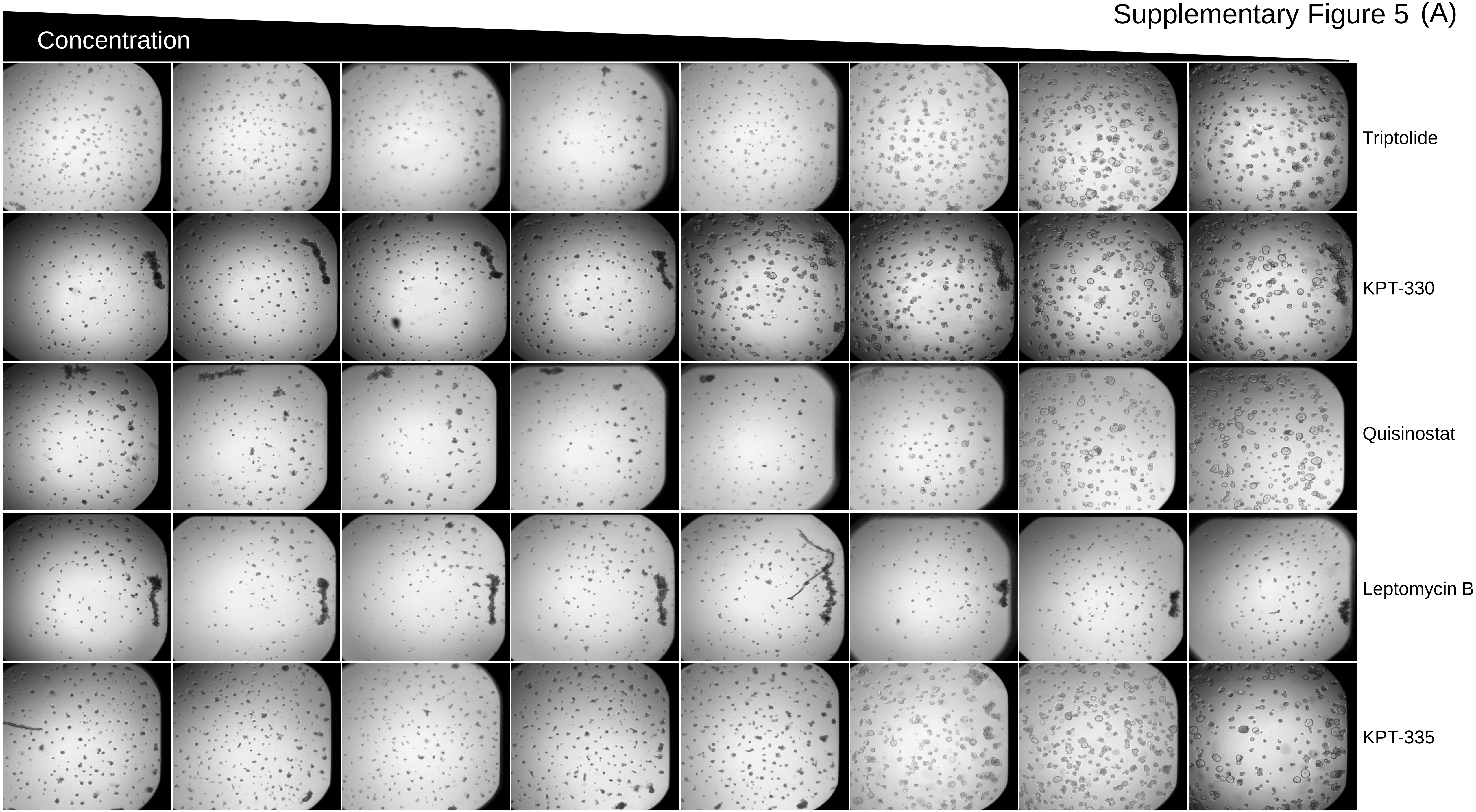

Supplementary Figure 5 (A) cont.

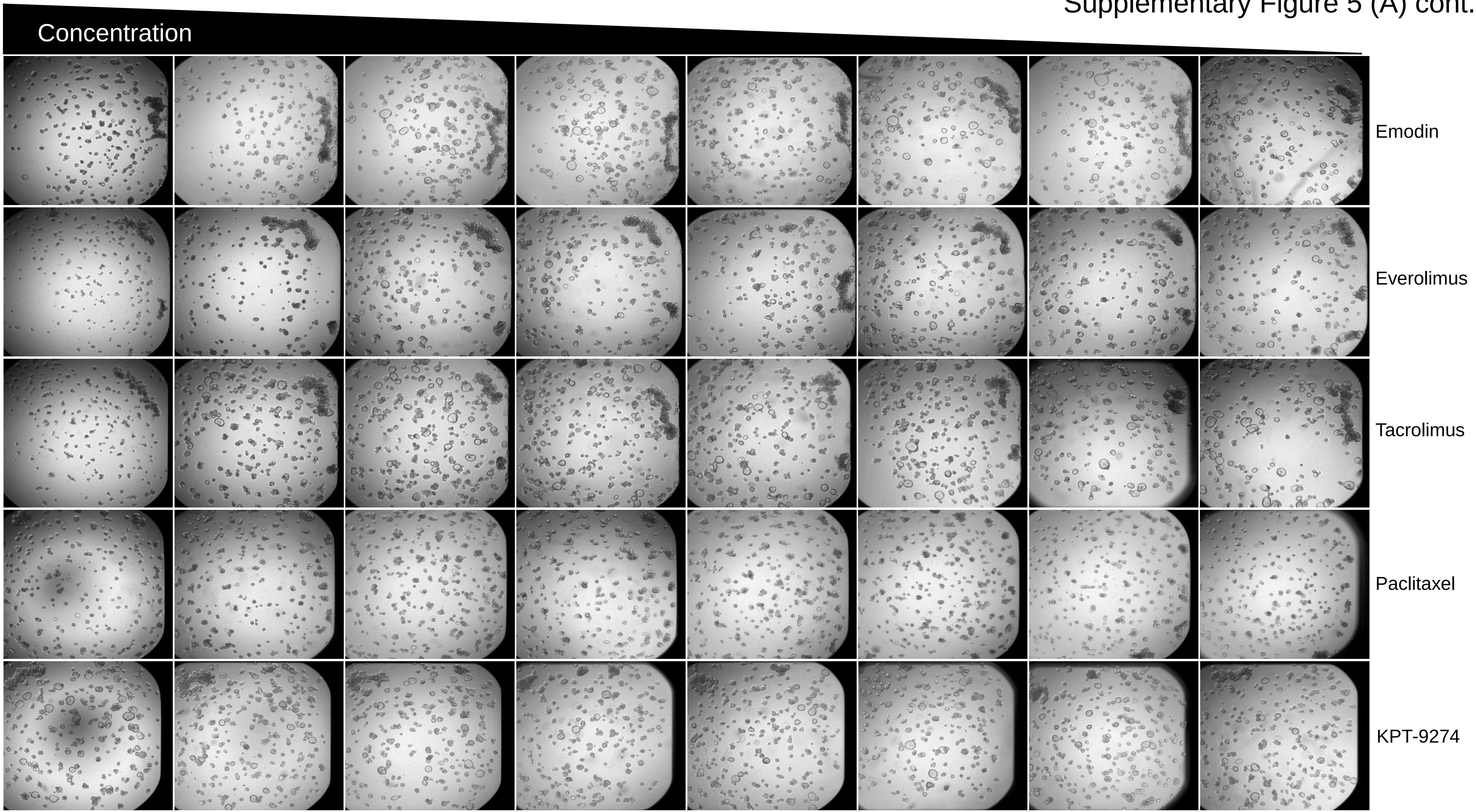

Supplementary Figure 5 cont.

(B)

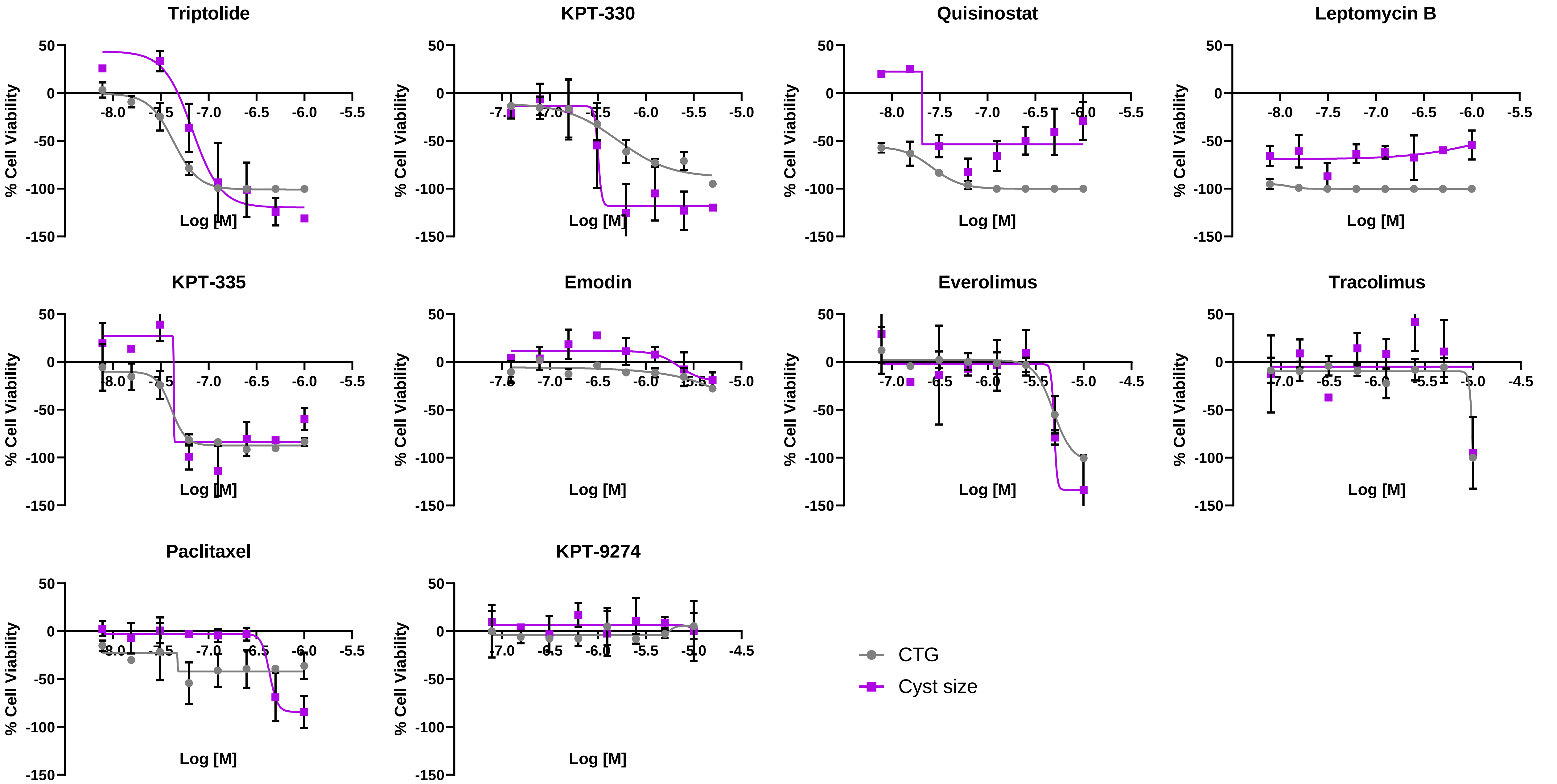

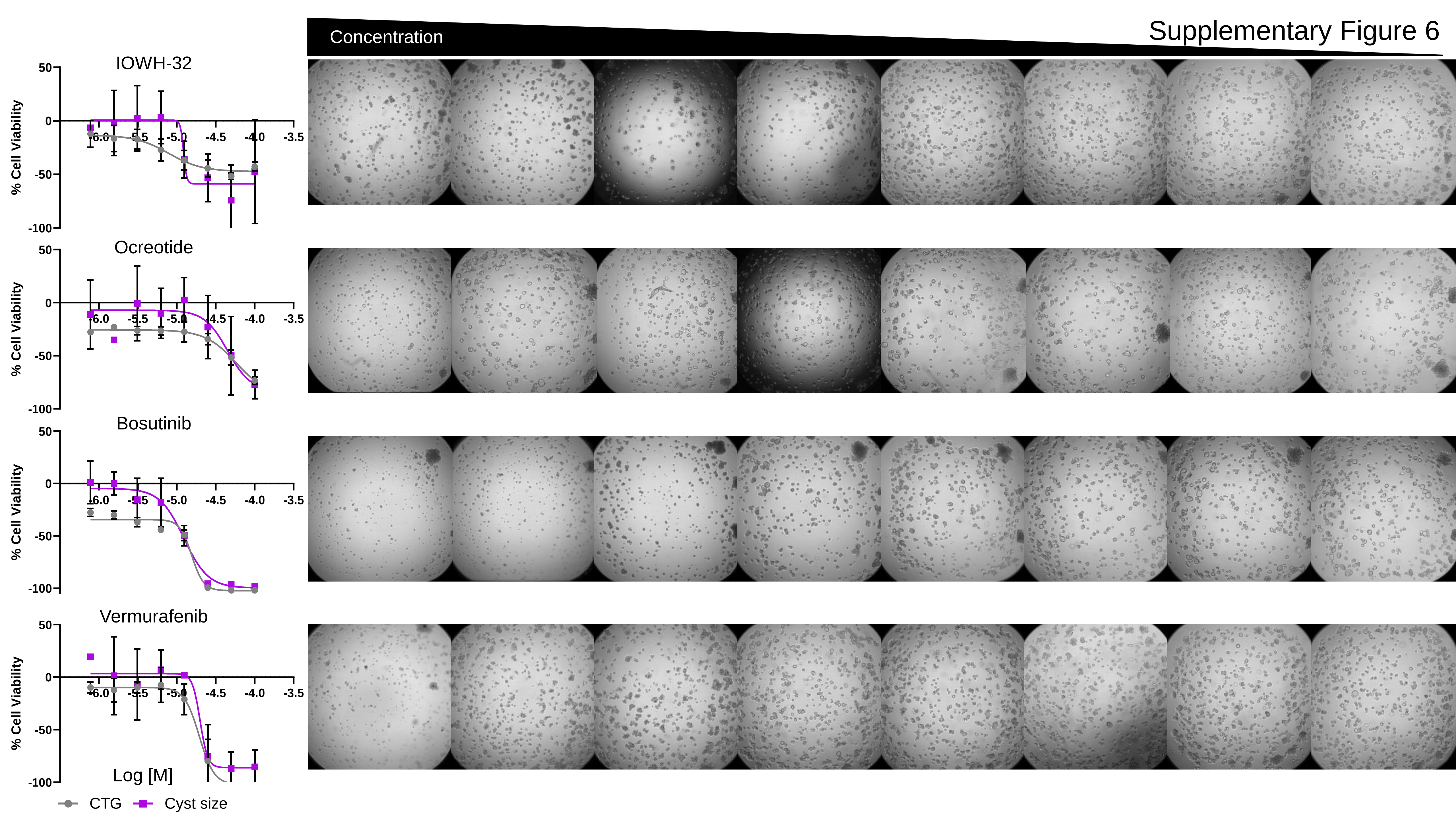

Concentration
Supplementary Figure 6
IOWH-32
Ocreotide
Bosutinib
Vermurafenib
% Cell Viability
50
0
-50
-100
-6.0
-5.5
-5.0
-4.5
-4.0
-3.5
Log [M]
CTG
Cyst size

Supplementary Figure 7

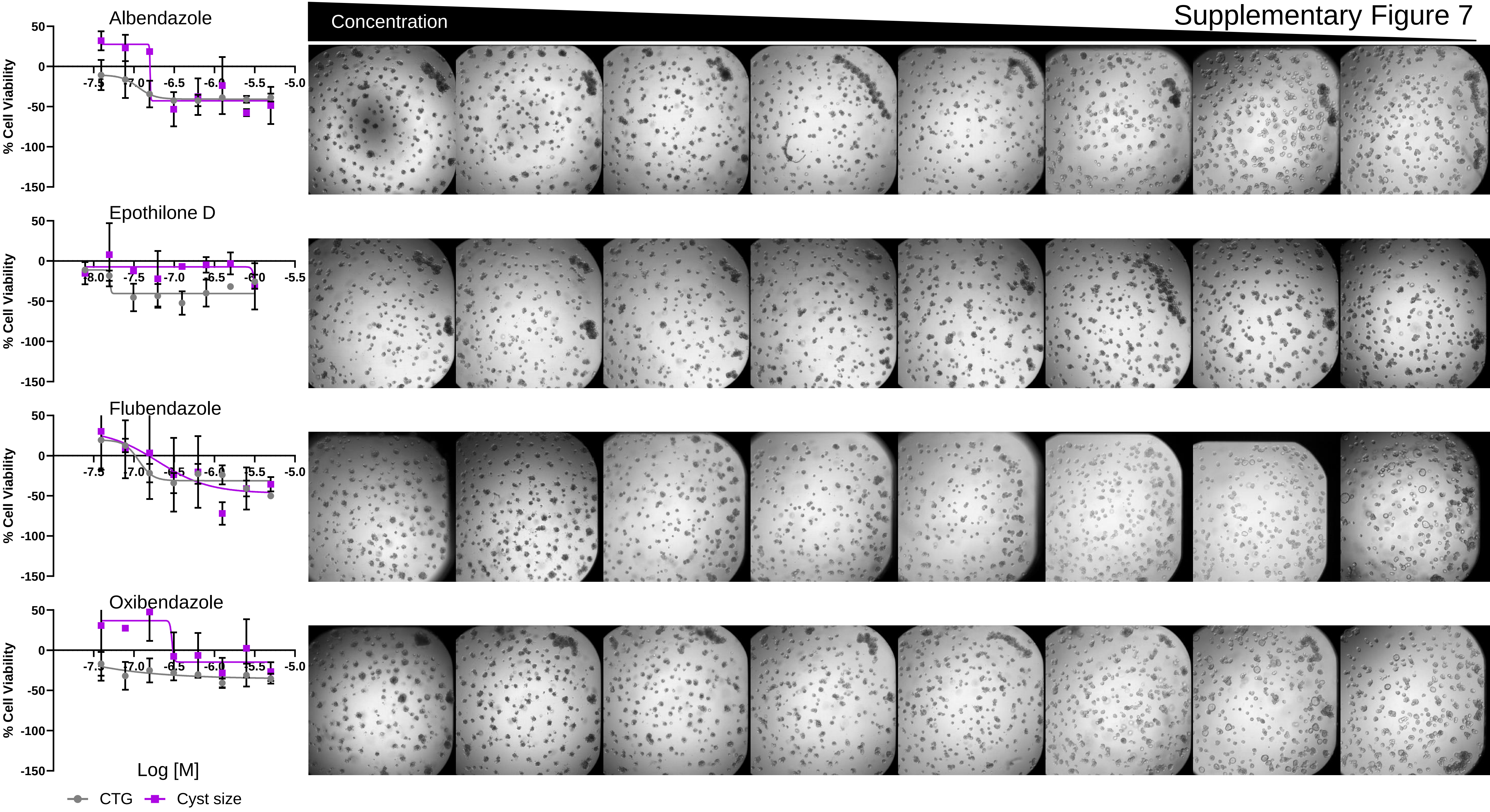

Supplementary Figure 8

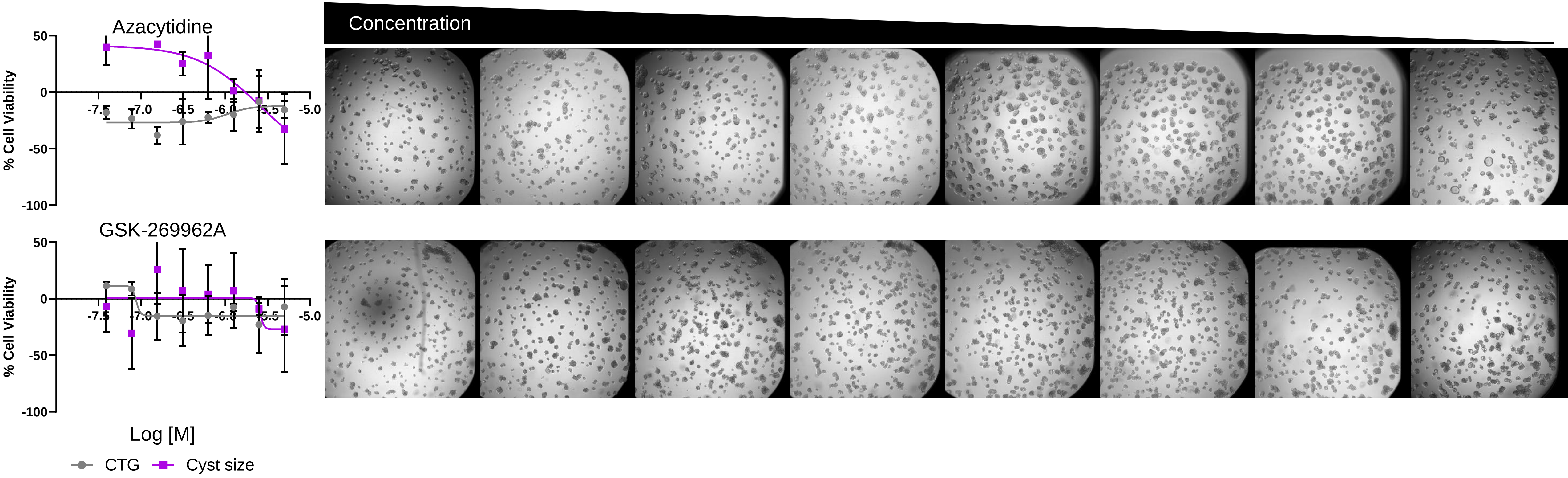

Supplementary Figure 9

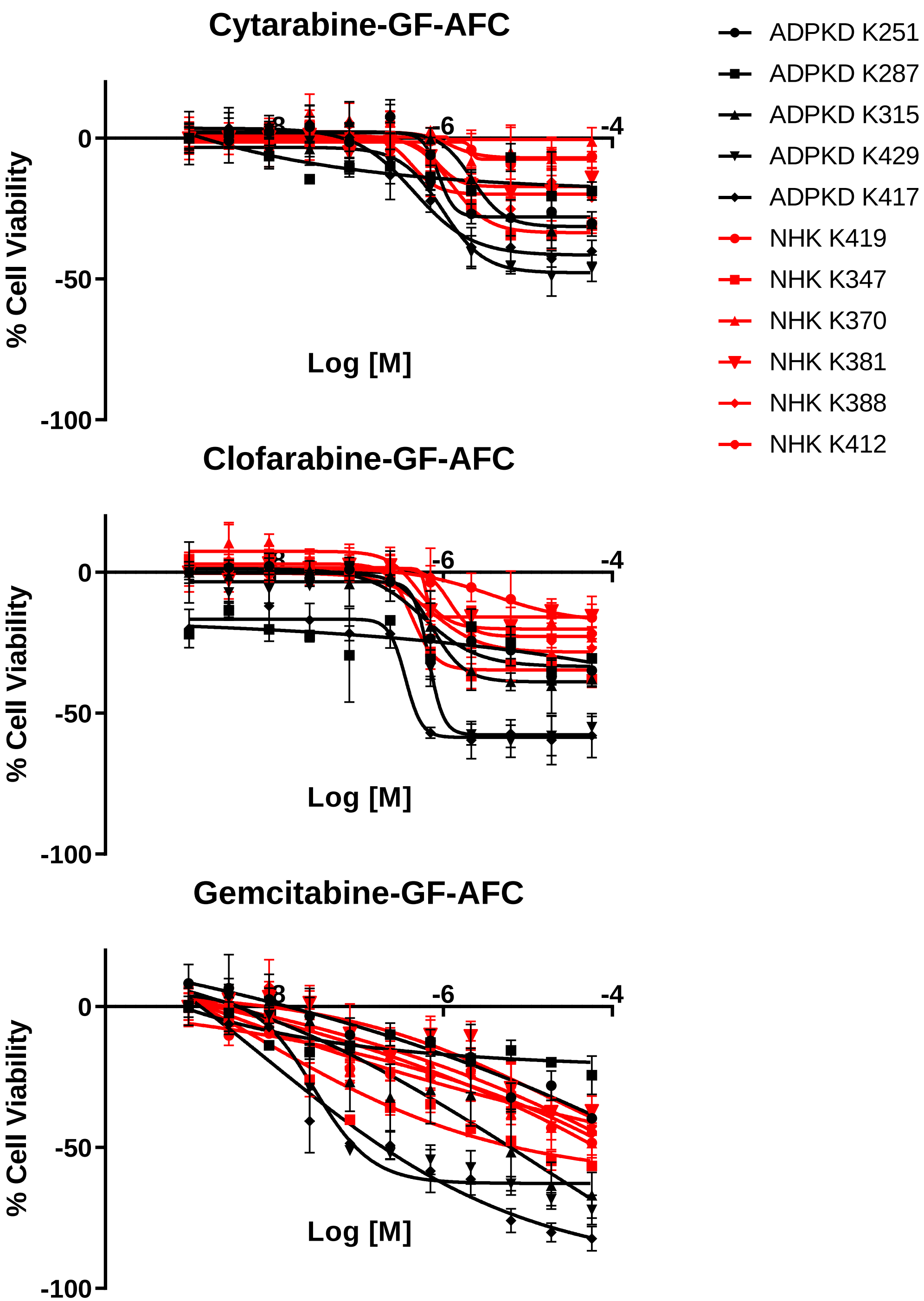

Supplement: Supplementary file 1 — Supplementary information. [file 41598_2020_61082_MOESM1_ESM.pdf]
